# Weak noncovalent interactions in 1,2,4-triazole-3-thione-linked adamantyl derivatives: experimental and computational insights into their potential as antiproliferative agents

**DOI:** 10.3389/fchem.2025.1691657

**Published:** 2025-11-27

**Authors:** Lamya H. Al-Wahaibi, Annesha Chakraborty, Hanan M. Hassan, Mohammed S. M. Abdelbaky, Santiago Garcia-Granda, Ali A. El-Emam, M. Judith Percino, Subbiah Thamotharan

**Affiliations:** 1 Department of Chemistry, College of Sciences, Princess Nourah bint Abdulrahman University, Riyadh, Saudi Arabia; 2 Biomolecular Crystallography Laboratory and DBT-Bioinformatics Center, School of Chemical and Biotechnology, SASTRA Deemed University, Thanjavur, India; 3 Department of Pharmacology and Biochemistry, Faculty of Pharmacy, Delta University for Science and Technology, Gamasa City, Egypt; 4 Department of Physical Chemistry, Faculty of Chemical Sciences, University of Salamanca, Salamanca, Spain; 5 Department of Physical and Analytical Chemistry, Faculty of Chemistry, Oviedo University-CINN, Oviedo, Spain; 6 Department of Medicinal Chemistry, Faculty of Pharmacy, Mansoura University, Mansoura, Egypt; 7 Unidad de Polímeros y Electrónica Orgánica, Instituto de Ciencias, Benemérita Universidad Autónoma de Puebla, Val3-Ecocampus Valsequillo, San PedroZacachimalpa, Mexico

**Keywords:** adamantyl, 1,2,4-triazole, weak noncovalent interactions, chalcogen bond, energy decomposition analysis

## Abstract

The synthesis, single-crystal X-ray structures, and antiproliferative activity of five adamantyl-linked 1,2,4-triazole-3-thione derivatives are presented. The crystal structures of the mono- and di-substituted chloro derivatives were analyzed in detail, revealing a variety of weak noncovalent interactions, including C–H···N, C–H···O, C–H···Cl, C–H···S, and π···π stacking, which contribute to the stability of their supramolecular assemblies. Notably, the crystal packing is also stabilized by σ-hole interactions, such as S···S and S···N chalcogen bonds, and by short, attractive Csp^3^–H···H–Csp^3^ contacts involving the adamantyl moieties. The nature and energetics of these interactions were investigated through a combination of Hirshfeld surface analysis, generalized Kohn-Sham energy decomposition analysis (GKS-EDA), and the quantum theory of atoms in molecules (QTAIM). The antiproliferative potential of these compounds was rationalized through molecular docking studies with urokinase plasminogen activator (uPA), which showed that the title compounds interact effectively with key catalytic residues. This work provides detailed insights into the role of weak noncovalent forces in the crystal engineering of adamantyl-triazole derivatives and supports their potential as uPA-directed anticancer agents.

## 1 Introduction

1,2,4-Triazoles are crucial class of heterocyclic compounds that hold a widespread range of pharmacological and chemotherapeutic activities (Aggarwal and Sumran, 2020; Ajmal et al., 2024; Parthasarathi and Kanagaraj, 2024). Several 1,2,4-triazole based drugs are currently used as efficient antifungal (Tratrat, 2020), antibacterial (Gao et al., 2019), anticancer (Rathod and Kumar, 2022), and antiviral (Bivacqua et al., 2023) agents. On the other hand, compounds containing an adamantane moiety were early identified as prominent pharmacophore position in the field of chemotherapy (Liu et al., 2011; Wanka et al., 2013). Several adamantane based drugs are currently employed as potent antiviral (Davies et al., 1964; Rosenthal et al., 1982), anticancer (Britten et al., 2017; Long et al., 2007), and anti-tubercular (Protopopova et al., 2005) agents. In a previous study, a series of adamantane-linked 1,2,4-triazole derivatives were reported to possess marked antimicrobial activities (Al-Omar et al., 2010). Based on the previously reported observations and our ongoing interest in the chemotherapeutic properties and structural characterization of adamantane-linked 1,2,4-triazole derivatives (Al-Wahaibi et al., 2021a; Al-Wahaibi et al., 2022a; Al-Wahaibi et al., 2022b; Al-Wahaibi et al., 2022c; Al-Wahaibi et al., 2023; Al-Omary et al., 2022; El-Emam et al., 2020; Warda et al., 2022), we report herein the single crystal X ray structures, electronic properties, and anti-proliferative activity of five adamantyl-linked 1,2,4-triazole derivatives.

The structural studies on adamantyl-linked 1,2,4-triazole derivatives is very limited (25 hits according to the Cambridge Structural Database (ver. 5.45) search) (Groom et al., 2016). Most of the structures reported from our laboratory. One of the structures, 2-(*N*-(2-fluorophenyl)aminomethyl)-5-adamantyl-2*H*-1,2,4-triazole-3(4*H*)-thione was reported from other group and the compound exhibited anti-cancer activity (Milošev et al., 2017). This is the first report on adamantyl-linked 1,2,4-triazole where a detailed energetics of molecular dimers of the title compounds and characterize the nature of various noncovalent interactions found in these dimers. The structures of the mono and di-substituted chlorine derivatives of the present work exhibit various types of weak noncovalent interactions, including C–H···N, C–H···O, C–H···Cl, C–H···S, C–H···π, π···π interactions. In addition to these interactions, one of the σ-hole interactions such as chalcogen bonding involving either S···S or S···N type interaction also contributed to the stability of these structures. One of the common features observed in the adamantyl derivative is a short and attractive weak H···H contact formed between adamantyl moieties within crystal structures or adamantyl with other groups having hydrogen atoms bound to C_sp3_ or C_sp2_ atoms. The energetics of molecular dimers was carried out using the generalized Kohn-Sham energy decomposition analysis (GKS-EDA) method (Su et al., 2020; Tang et al., 2021). Furthermore, the nature and strength of noncovalent interactions observed within the dimers of these structures were characterized using Bader’s QTAIM framework.

The anti-proliferative activity of the title compounds was reinforced by molecular docking with urokinase plasminogen activator (uPA), a serine protease whose overexpression is closely associated with tumor cell migration, invasion, proliferation, progression, and metastasis (Reuning et al., 1998). Targeting uPA therefore represents an attractive therapeutic approach. Crystallographic analysis of uPA B-chain with the most active *N*-(1-adamantyl)-*N*’-(4-guanidinobenzyl)urea derivative provided insight into binding modes (Sperl et al., 2000). Molecular docking further showed that the title compounds interact with key catalytic residues of uPA, thereby rationalizing their biological activity and supporting their potential as uPA-directed anticancer agents.

## 2 Materials and methods

### 2.1 Synthesis and crystallization

**SCHEME 1:**
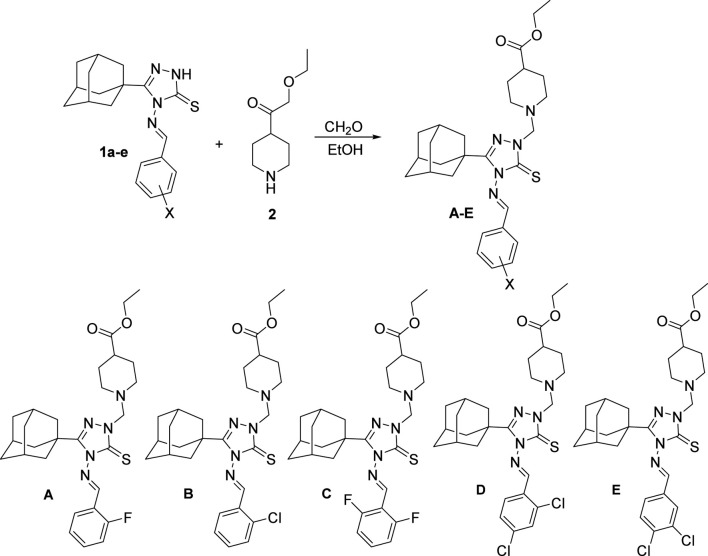
The synthetic pathway and structures of the investigated compounds **(A–E)**. Ethyl 1-{[3-(adamantan-1-yl)-4-{[(2-fluorophenyl)methylidene]amino}-5-sulfanylidene-4,5-dihydro-1*H*-1,2,4-triazol-1-yl]methyl}piperidine-4-carboxylate **(A)** Mol. Formula (Mol. Weight): C_28_H_36_FN_5_O_2_S (525.69). Yield: 72%. Melting point: 134 °C–136 °C (uncorrected). Ethyl 1-{[3-(adamantan-1-yl)-4-{[(2-chlorophenyl)methylidene]amino}-5-sulfanylidene-4,5-dihydro-1*H*-1,2,4-triazol-1-yl]methyl}piperidine-4-carboxylate **(B)** Mol. Formula (Mol. Weight): C_28_H_36_ClN_5_O_2_S (542.14). Yield: 75%. Melting point: 158 °C–160 °C (uncorrected). Ethyl 1-{[3-(adamantan-1-yl)-4-{[(2,6-difluorophenyl)methylidene]amino}-5-sulfanylidene-4,5-dihydro-1*H*-1,2,4-triazol-1-yl]methyl}piperidine-4-carboxylate **(C)** Mol. Formula (Mol. Weight): C_28_H_35_F_2_N_5_O_2_S (543.68). Yield: 69%. Melting point: 148 °C–150 °C (uncorrected). Ethyl 1-{[3-(adamantan-1-yl)-4-{[(2,4-dichlorophenyl)methylidene]amino}-5-sulfanylidene-4,5-dihydro-1*H*-1,2,4-triazol-1-yl]methyl}piperidine-4-carboxylate **(D)** Mol. Formula (Mol. Weight): C_28_H_35_Cl_2_N_5_O_2_S (576.58). Yield: 68%. Melting point: 129 °C–131 °C (uncorrected). Ethyl 1-{[3-(adamantan-1-yl)-4-{[(3,4-dichlorophenyl)methylidene]amino}-5-sulfanylidene-4,5-dihydro-1*H*-1,2,4-triazol-1-yl]methyl}piperidine-4-carboxylate **(E)** Mol. Formula (Mol. Weight): C_28_H_35_Cl_2_N_5_O_2_S (576.58). Yield: 71%. Melting point: 186 °C–188 °C (uncorrected).

The investigated compounds **A-E** were prepared via reaction of the corresponding 5-(adamantan-1-yl)-4-(haloarylideneamino)-2,4-dihydro-3*H*-1,2,4-triazole-3-thione **1a-e** with ethyl 4-piperidinecarboxylate and 37% formaldehyde solution, in ethanol to yield the target compounds as previously reported (Al-Omar et al., 2010). Pure single crystals were obtained by slow evaporation of CHCl_3_:EtOH (1:1, v/v) solution at room temperature to yield the compounds as colorless prism crystal (Scheme 1).

### 2.2 Single-crystal X-ray diffraction

X-ray intensity data for crystals of compounds **A-E** were collected at room temperature using an Xcalibur Ruby Gemini diffractometer with a single-wavelength X-ray source (Cu Kα radiation: λ = 1.54184 Å). Pre-experiment setup, data collection, data reduction, and analytical absorption correction (Clark and Reid, 1995) were carried out using the CrysAlisPro software suite (ver. 1.171.37.35, Oxford Diffraction, 2014). The structures were solved using the SIR-2011 program (Burla et al., 2012), and structural refinement was performed using the SHELXL 2018/3 package (Sheldrick, 2015). All hydrogen atoms were positioned in idealized locations (C−H = 0.93–0.98 Å) and constrained to ride on their parent atoms with *U*_iso_(H) = 1.2*U*_eq_(C). For methyl groups, hydrogen atoms were placed in ideal geometry (C−H = 0.96 Å) with *U*_iso_(H) = 1.5*U*_eq_(C) and allowed to rotate freely about the C−C bond. Due to the poor quality of crystals for compounds **A** and **C**, well-refined models could not be obtained, so these were excluded from further structural analysis. Nevertheless, X-ray analysis confirmed the structures of compounds **A** and **C** (Supplementary Table S1).

### 2.3 Hirshfeld surface analysis and 2D-fingerprint plots

We performed Hirshfeld surface analysis for compounds **B, D,** and **E** using their crystallographic information files (CIF) and the CrystalExplorer-21.5 program (Spackman et al., 2021). From this analysis, 2D fingerprint plots were generated to quantify the contributions of intermolecular interactions within the structures, with the aim of understanding the effects of mono- and dichloro substitutions. Deformation density calculations were carried out at the HF/6-311G (d,p) level of theory (Hehre et al., 1972) using the CrystalExplorer program.

### 2.4 DFT calculations

All gas-phase geometry optimization were performed with the Gaussian 09 program (Frisch et al., 2013) at the M06-2X/def2-TZVP level of theory (Weigend and Ahlrichs, 2005; Zhao and Truhlar, 2008), including Grimme’s D3 empirical dispersion correction (Grimme et al., 2010; Grimme et al., 2011). Vibrational frequency calculations at the same level confirmed all optimized structures as true energy minima. For the molecular dimers identified in the crystal structures of compounds **B**, **D**, and **E**, the C–H bond distances were first normalized to typical neutron diffraction values (1.083 Å) (Groom et al., 2016). The interaction energies of these dimers were then analyzed using generalized Kohn-Sham energy decomposition analysis (GKS-EDA) in the XEDA program (Su et al., 2020; Tang et al., 2021) at the

ωB97X-D/def2-TZVP level (Chai and Head-Gordon, 2008; Weigend and Ahlrichs, 2005). This method partitions the total interaction energy (Δ*E* ^total^) into four key components: electrostatic energy (Δ*E* ^ele^), exchange-repulsion energy (Δ*E* ^exrep^), polarization energy (Δ*E* ^pol^), and correlation/dispersion energy (Δ*E* ^corr/disp^), where the latter term includes both short- and long-range dispersion contributions (Xu et al., 2023). To further characterize the intermolecular interactions, the dimers were also analyzed using the QTAIM (Bader, 1994) with the AIMALL program (Keith, 2019).

### 2.5 Molecular docking

The X-ray structures of compounds **B**, **D**, and **E**, along with the optimized structures of **A** and **C**, were used for molecular docking studies. The protein target, urokinase plasminogen activator B-chain (uPA), was obtained from the Protein Data Bank (PDB ID: 1EJN), with only chain A considered for docking. Molecular docking simulation was performed using the CB-Dock2 server (Liu et al., 2022), which employs the Vina docking code to generate docking scores and poses (Eberhardt et al., 2021). uPA was co-crystallized with the inhibitor *N*-(1-adamantyl)-*N*’-(4-guanidinobenzyl)urea (compound ID: AGB), which was also re-docked using the same program to verify reproducibility. The top-scoring pose was then subjected to protein-ligand interaction analysis with the PLIP web server (Adasme et al., 2021).

## 3 Results and discussion

In this work, we present the crystal structures of three 1,2,4-triazole-3-thione-linked adamantyl derivatives, along with two related compounds that were modelled and fully optimized in the gas phase. Their antiproliferative activity was evaluated, and *in vitro* findings were further supported by molecular docking and molecular dynamics simulations. These derivatives share a central 1,2,4-triazole-3-thione core, with three distinct chemical scaffolds attached: an adamantane cage, an ethoxycarbonyl-piperidyl group, and a substituted benzylideneamino group.

The compounds differ in the type of halogen substitution (fluorine or chlorine) in the benzylidene ring. Two are monosubstituted, while the remaining three feature di-substitutions. All five compounds were successfully crystallized for X-ray analysis, although the quality of the X-ray data varied. The chloro-substituted compounds provided high-quality data suitable for detailed structural analysis, whereas the fluoro-substituted derivatives yielded lower-quality data. Multiple attempts to obtain high-quality crystals and X-ray data for the fluoro derivatives were unsuccessful. Crystal data and structure refinement parameters for compounds **B**, **D**, and **E** are summarized in Table 1, while the corresponding details for the fluoro-substituted compounds are provided in Supplementary Table S1. The unit-cell dimensions of compounds **A**, **B**, and **C** are comparable, and they exhibit similar packing features in the solid state (Supplementary Figure S1).

**TABLE 1:** Crystal data and structure refinement parameters for compounds **B, D and E**.

Identification code	B	D	E
Empirical formula	C_28_H_36_N_5_O_2_SCl	C_28_H_35_N_5_O_2_SCl_2_	C_28_H_35_N_5_O_2_SCl_2_
Formula weight	542.13	576.57	576.57
Temperature/K	293 (2)	293 (2)	293 (2)
Crystal system	Triclinic	Monoclinic	Triclinic
Space group	*P*-1	*P*2_1_/c	*P*-1
a/Å	7.5711 (7)	13.8223 (6)	9.8923 (5)
b/Å	13.7132 (13)	7.4117 (3)	11.8359 (8)
c/Å	13.7884 (11)	28.4957 (12)	13.4614 (9)
α/°	88.943 (7)	90	103.213 (5)
β/°	81.707 (7)	100.786 (4)	107.620 (5)
γ/°	87.026 (7)	90	91.152 (5)
Volume/Å^3^	1414.6 (2)	2,867.7 (2)	1455.62 (16)
Z	2	4	2
ρ_calc_g/cm^3^	1.273	1.335	1.315
μ/mm^-1^	2.152	2.994	2.949
F (000)	576.0	1216.0	608.0
Crystal size/mm^3^	0.11 × 0.07 × 0.06	0.35 × 0.07 × 0.05	0.25 × 0.12 × 0.11
Radiation	CuKα (λ = 1.54184)	CuKα (λ = 1.54184)	CuKα (λ = 1.54184)
2Θ range for data collection/°	6.454 to 130.154	6.316 to 151.612	7.108 to 151.232
Index ranges	−8 ≤ h ≤ 8, −16 ≤ k ≤ 16, −16 ≤ l ≤ 16	−17 ≤ h ≤ 16, −8 ≤ k ≤ 9, −35 ≤ l ≤ 35	−12 ≤ h ≤ 12, −14 ≤ k ≤ 14, −16 ≤ l ≤ 16
Reflections collected	19,495	22,603	22,559
Independent reflections	4,812 [R_int_ = 0.1034, R_sigma_ = 0.0950]	5,864 [R_int_ = 0.1024, R_sigma_ = 0.1031]	5,947 [R_int_ = 0.0523, R_sigma_ = 0.0495]
Data/restraints/parameters	4,812/1/335	5,864/1/337	5,947/0/343
Goodness-of-fit on F^2^	1.019	1.031	1.016
Final R indexes [I ≥ 2σ (I)]	R_1_ = 0.0660, wR_2_ = 0.1480	R_1_ = 0.0691, wR_2_ = 0.1423	R_1_ = 0.0562, wR_2_ = 0.1362
Final R indexes [all data]	R_1_ = 0.1428, wR_2_ = 0.1926	R_1_ = 0.1561, wR_2_ = 0.1926	R_1_ = 0.1070, wR_2_ = 0.1670
Largest diff. Peak/hole/e Å^-3^	0.30/-0.33	0.67/-0.41	0.60/-0.28

**FIGURE 1:**
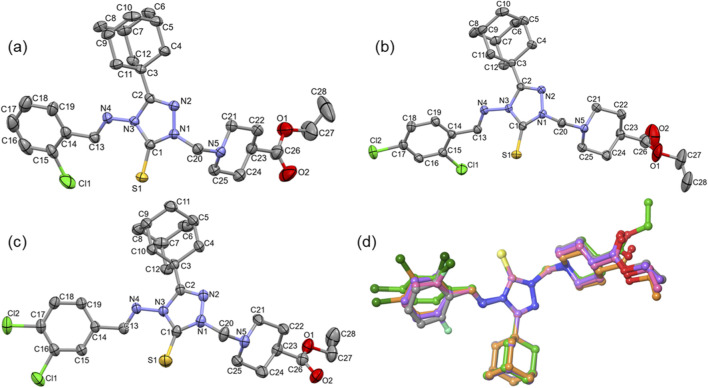
**(a–c)** ORTEP representation with atomic labelling scheme for compounds **B**, **D**, and **E**. **(d)** Structural superimposition of compounds **A-E**. H atoms have been omitted for clarity in all representations.

X-ray analysis reveals that compound **D** crystallizes in the monoclinic system (space group *P*2_1_/c), while compounds **B** and **E** crystallize in the triclinic system (space group *P*-1). ORTEP representations of these compounds are shown in Figures 1a–c. Structural superimposition of compounds **B**, **D** and **E** highlights a strong alignment of the 1,2,4-triazole-3-thione core, adamantane cage and piperidine moieties (Figure 1d). However, the substituted benzylidene ring exhibits some rotational distortion, and deviations are also observed in the orientation of the ethoxycarbonyl group. Interestingly, compounds **B** and **E** share a similar ethoxycarbonyl orientation, whereas compound **D** adopts a distinct conformation. In all five compounds, the fused-ring systems of the adamantyl moiety and piperidine ring adopt a chair conformation.

### 3.1 Hirshfeld surface analysis and 2D-fingerprint plots

**FIGURE 2:**
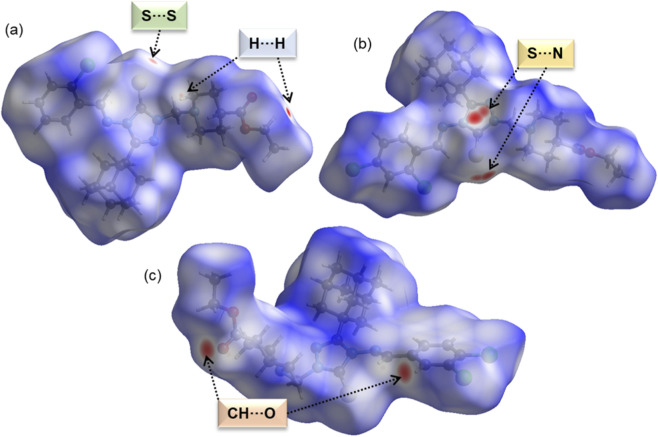
Hirshfeld surfaces mapped over the normalized distance (*d*_norm_) for **(a)** compound **B**, **(b)** compound **D**, and **(c)** compound **(E)**. Red spots indicating specific short contacts are labelled.

Hirshfeld surface analysis reveals key short contacts that likely contribute to the stability of each crystal structure (Figures 2a–c). In compound **B**, a small red spot marks a short S···S contact, indicating a chalcogen bond. Additionally, short H···H contacts appear as small red spots, particularly around the surface of the piperidine ring and terminal methyl group. In compound **D**, double red spots near thione group and over the triazole ring surface suggest S···N/π chalcogen bonding. For compound **E**, red spots highlight intermolecular C–H···O interactions. These findings indicate that such contacts play a crucial role in stabilizing the crystal structures.

**FIGURE 3:**
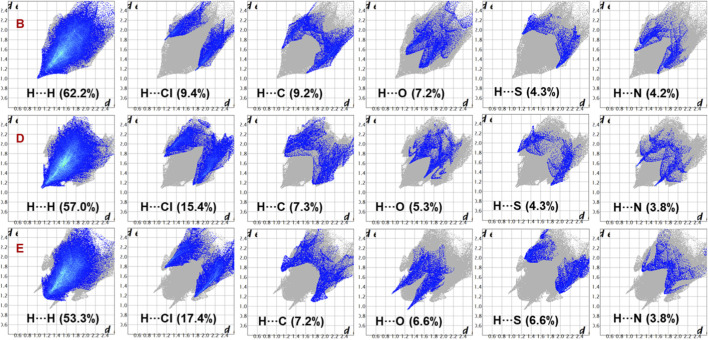
Decomposed 2D fingerprint plots showing the relative contributions of various intermolecular interactions in the crystal structures of compounds **B**, **D**, and **E**.

2D fingerprint plots reveal the relative contributions of various intermolecular interactions in crystal structures (Figure 3). Notable differences in H···H interactions were observed, with a higher contribution in the monochloro-substituted compound, as expected for compound **B**. In compound **B**, the H···H contact distance (*d*_e_ + *d*_i_) is approximately 2.0 Å, whereas in the other two compounds, this distance is slightly longer. Overall, H···H interactions account for 53.3%–62.2% of the total Hirshfeld surface area. Similarly, the contribution of H···Cl contacts increases slightly when the compounds contain a dichloro-substituted phenyl ring, ranging from 9.4% to 17.4% of the crystal packing. The nature of these H···Cl contacts also varies: in compound **B**, the shortest H···Cl contact is about 3.0 Å, whereas in the dichloro derivatives, it is slightly shorter, indicating its role in the stabilizing dichloro structures. For H···C contacts, which indicate intermolecular C–H···π interactions, the shortest distance is 2.7 Å in the monochloro derivative, while in the dichloro derivatives, it exceeds 2.7 Å, suggesting a weaker interaction. The contribution of H···C contacts remains similar across all three derivatives, but is slightly higher in compound **B**.

While H···O interactions show comparable contributions across all three compounds, their distances differ. In compound **B**, the shortest distance of is 2.7 Å, compared to 2.9 Å in compound **D** and 2.2 Å in compound **E**. This suggests that H···O contacts significantly enhance the stability of compound **E**. The H···S contact distance is 2.9 Å in compound **B** and longer in the other two structures, indicating a weaker interaction. Meanwhile, H···N contacts contribute 3.8%–4.2% of the crystal packing, with the shortest contact distances being 2.7 Å, 2.6 Å, and 3.0 Å in compounds **B**, **D**, and **E**, respectively. This pattern suggests that H···N contacts play a key role in stabilizing the crystal structure of compound **D**. As shown in Supplementary Figure S2, the presence of π-stacking interactions in these compounds is confirmed by the shape index diagram, in which red-blue triangles are observed over the phenyl ring. The contributions of these contacts are 1.4% (compound **B**), 1.7% (compound **D**) and 2.1% (compound **E**). The sulfur-based contacts, such as S···S (0.5%), S···N (1.3%) and S···C (0.4%) are observed in compound **B**. The first contact can be described as a chalcogen bond, while the latter two correspond to lone-pair-π contacts. Similarly, compound **D** also exhibit S···N contact, contributing 1.2% of the total HS area, which also represents a chalcogen bond. Although these contributions are significant, they still play an important role in stabilization.

### 3.2 Molecular dimers of compound B and their energetics

**FIGURE 4:**
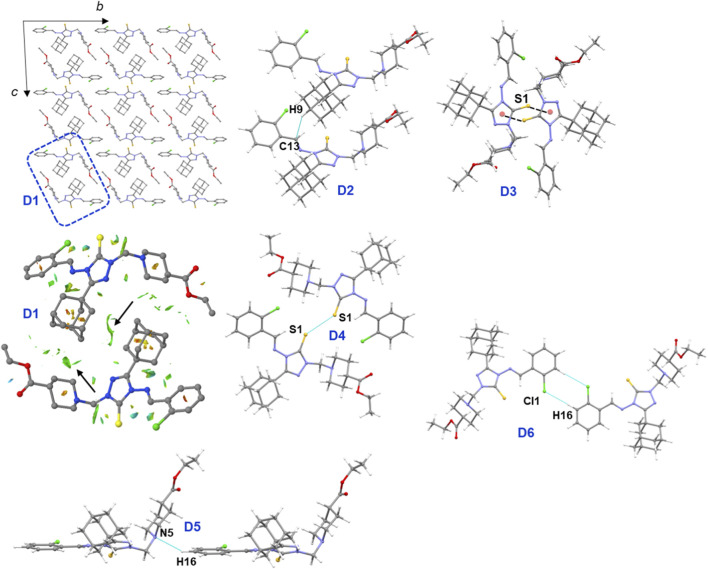
Columnar molecular arrangement in the solid state of compound **B**, and various molecular dimers observed in this structure are shown. In dimer D1, the green patches indicate short H···H contacts between the adamantane moieties and between the adamantane and piperidine moieties.

In the crystalline state, molecules of compound **B** arrange in a columnar fashion (Figure 4) to form six distinct molecular dimers. These dimers are stabilized by various non-covalent interactions, including short Csp^3^–H···H–Csp^3^ contacts, a σ-hole chalcogen bond (S···S), C–H···N/Cl/π interactions, and lone pair-π interactions. The geometric parameters and intermolecular interaction energies for these dimers, which range from −14.23 to −2.14 kcal mol^−1^, are presented in Table 2.

**TABLE 2:** Intermolecular interactions observed in various dimers of compound **B**, with decomposed energy components expressed in kcal mol^−1^.

Motif	Symmetry	Interactions	Geometry^a^ H···A (Å) ∠D–H···A (°)	Δ*E* ^ele^	Δ*E* ^exrep^	Δ*E* ^pol^	Δ*E* ^corr/disp^	Δ*E* ^total^
D1	1−*x*, 1−*y*, 1−*z*	Csp^3^–H···H–Csp^3^	<2.40	−3.68	11.34	−0.05	−21.84	−14.23
D2	1 + *x*, *y*, *z*	C9–H9···C13(π)	2.75, 147	−4.08	10.52	−0.68	−19.86	−14.11
D3	1−*x*, 1−*y*, −*z*	S1···*Cg*1	3.460(2), 92.99(16)	−8.22	15.18	−1.99	−18.47	−13.50
D4	−*x*,1−*y*, −*z*	C1–S1···S1	3.510(2), 138.4(2)	−1.78	7.83	−0.77	−9.65	−4.38
D5	*x*, −1 + *y*, *z*	C17–H17···N5	2.71, 140	−1.60	3.88	−0.46	−5.65	−3.83
D6	−*x*, −*y*, −*z*	C16–H16···Cl1	2.98, 156	−1.60	2.12	−0.52	−2.15	−2.14

^a^ Neutron diffraction values are given for all D–H⋅⋅⋅A interactions. *Cg*1 represents the centroid of the triazole ring.

As shown in Figure 4, the core structural motif, D1, is formed by short Csp^3^–H···H–Csp^3^ contacts between adjacent adamantane and adamantane-piperidine group. The NCI plot reveals green patches between the interacting moieties, which confirms the presence of these weak stabilizing interactions. The total energy for this dimer is calculated to be −14.23 kcal mol^−1^, with dispersion energy contributing the majority (85%) to its stabilization. This non-electrostatic origin of Csp^3^–H···H–Csp^3^ contacts in adamantane derivatives has been reported in previous studies (Al-Omary et al., 2022; Al-Wahaibi et al., 2021a; Al-Wahaibi et al., 2022a; Al-Wahaibi et al., 2023; Bicknell et al., 2024; El-Emam et al., 2020).

The second strongest dimer, D2, with a Δ*E* ^total^ value of −14.11 kcal mol^−1^, is stabilized by an intermolecular C–H···C(π) interaction that links neighbouring molecules into a chain. The strength of this dimer is comparable to that of dimer D1. As expected, the dispersion energy is the dominant contributor to the stabilization of dimer D2, accounting for 81% of the total stabilization energy. The next strongest dimer, D3 (Δ*E* ^total^ = −13.50 kcal mol^−1^), is stabilized by lone pair···π interactions involving the S1 atom and the π-center of the central triazole ring. This dimer is formed between molecules related by a center of inversion symmetry, and dispersion energy contributes 64% to its stabilization.

Of particular interest is the σ-hole interaction (chalcogen bond) formed between adjacent molecules via an S···S contact in dimer D4 (Δ*E* ^total^ = −4.38 kcal mol^−1^). Energy decomposition analysis reveals that this dimer is predominantly stabilized by dispersion energy, which contributes 79% of the total stabilization energy. This interaction was further characterized using a deformation electron density map. The σ-hole interactions (chalcogen, halogen, tetrel, etc.) are routinely characterized using deformation electron density maps, which highlight electron-deficient (donor) and electron-rich (acceptor) regions to help understand the formation of these interactions (Khan et al., 2016; Thomas et al., 2022). The map clearly shows an electron-deficient region observed at the tip of 1 S atom projected toward the electron-rich (lone-pair) region of neighbouring S atom, confirming the presence of a chalcogen bond that contributes to the stabilization of the structure.

**FIGURE 5:**
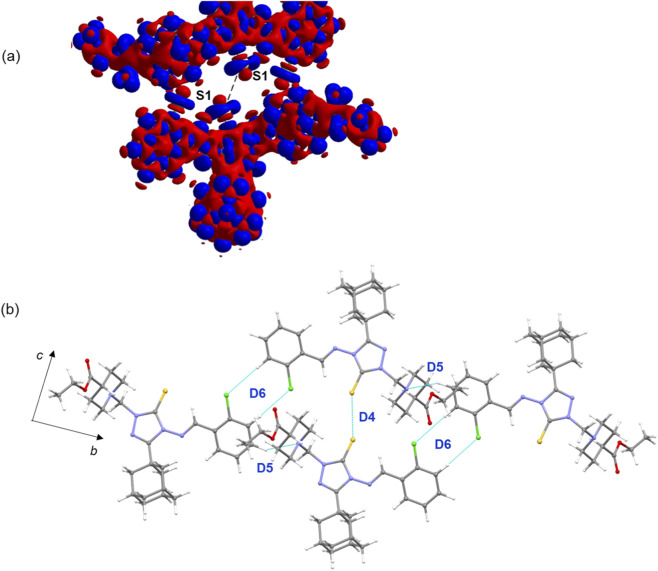
**(a)** Deformation electron density map highlighting a chalcogen bond between sulfur atoms. The red and blue surfaces represent regions of charge depletion and charge concentration, respectively. **(b)** Supramolecular chain constructed from dimeric motifs D4–D6 in the crystal structure of compound **B**.

Dimer D5 is stabilized by an intermolecular C–H···N interaction involving the piperidine N and the substituted phenyl ring, with a Δ*E* ^total^ value of −3.83 kcal mol^−1^. Dispersion energy contributes 73% to its stabilization. The sixth dimer, D6, is stabilized by an intermolecular C–H···Cl interaction (Δ*E* ^total^ = −2.14 kcal mol^−1^). This interaction occurs between molecules related by a center of inversion symmetry and gives rise to an $R_{2}^{2}$ (8) motif, one of the most recurring patterns observed in organic crystals (Esterhuysen, 2024). Dispersion (50%) and electrostatic (50%) energies contribute equally to the stabilization of this cyclic dimer. Furthermore, the last three dimers (D4, D5 and D6) generate a supramolecular chain, as shown in Figure 5.

### 3.3 Molecular dimers of compound D and their energetics

**FIGURE 6:**
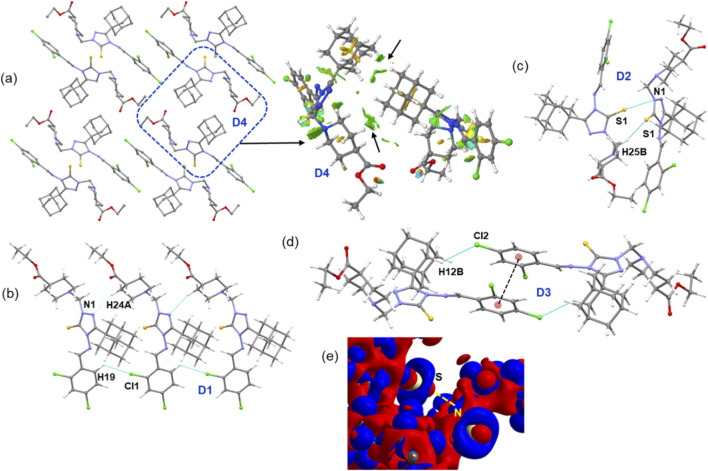
**(a)** Columnar crystal packing of compound **D** viewed along the crystallographic *ac* plane, with the core structural motif formed by dimer D4 highlighted in a box. Green patches indicate weak and short H···H contacts. **(b-d)** Molecular dimers of compound **D** are stabilized by various noncovalent interactions. **(e)** The deformation electron density map reveals a chalcogen bond.

The crystal packing of compound **D** closely resembles that of compound **B**, with notable differences in the molecular dimers. In the solid state, four energetically significant molecular dimers are observed, and their geometrical parameters along with energy components are listed in Table 3. The crystal packing and molecular dimers of compound **D** are illustrated in Figure 6a. Similar to compound **B**, the core motif (D4) in compound **D** is stabilized by short Csp^3^–H···H–Csp^3^ contacts, with a Δ*E* ^total^ value of −7.19 kcal mol^−1^. These interactions involve adjacent adamantane groups as well as adamantane-piperidine groups. The NCI plot reveals green patches in the interacting regions, confirming the weak but attractive nature of these forces. Energy decomposition analysis indicates that the dispersion energy component (Δ*E* ^corr/disp^) contributes about 85% to the stabilization of dimer D4.

**TABLE 3:** Intermolecular interactions observed in various dimers of compound **D**, with decomposed energy components expressed in kcal mol^−1^.

Motif	Symmetry	Interactions	Geometry^a^ H···A (Å) ∠D–H···A (°)	Δ*E* ^ele^	Δ*E* ^exrep^	Δ*E* ^pol^	Δ*E* ^corr/disp^	Δ*E* ^total^
D1	*x*, 1 + *y*, *z*	C24–H24A···N2	2.66, 163	−4.37	12.52	−0.63	−22.61	−15.08
		C19–H19···Cl1	2.90, 124					
D2	1−*x*, −½+*y*, ½−z	C25–H25B···S1	2.94, 139	−7.99	19.59	−2.29	−22.89	−13.59
		C1–S1···N1	3.161(2), 170.96 (2)					
D3	1−*x*, 1−*y*, 1−*z*	C12–H12B···Cl2	2.96, 136	−4.29	11.45	−1.34	−16.97	−11.15
		*Cg*1···*Cg*1	3.681 (3)					
D4	−*x*, ½+*y*, ½−*z*	Csp^3^–H···H–Csp^3^	<2.40	−1.61	5.05	−0.22	−10.42	−7.19

^a^ Neutron diffraction values are given for all D–H⋅⋅⋅A interactions. *Cg*1 represents the centroid of the phenyl ring.

Dimer D1 is stabilized by intermolecular C–H···N and C–H···Cl interactions, with a Δ*E* ^total^ value of −15.08 kcal mol^−1^ (Figure 6b). These interactions link neighbouring molecules into a chain running parallel to the crystallographic *b* axis. This stabilization is predominantly driven by dispersion energy, contributing 82%. Dimer D2 is stabilized by an intermolecular C–H···S interaction and additionally features a chalcogen bond (S···N) between the thione S atom and a nitrogen atom of the triazole ring (Figure 6c). This chalcogen bond differs from that observed in compound **B**. Energetic characterization of such chalcogen bonds, particularly in organic molecular solids containing a 1,2,4-triazole ring, has been reported previously (Al-Wahaibi et al., 2020; Al-Wahaibi et al., 2021b; Al-Wahaibi et al., 2022b). For this dimer, dispersion energy contributes 69% to the stabilization. As shown in Figure 6e, the deformation electron density map reveals a chalcogen bond between a sulfur atom and a triazole nitrogen atom. The map shows a region of charge concentration located around the nitrogen atom, which is directed toward a region of charge depletion on the sulfur atom.

The energy of molecular dimer D3 is also significant, with a Δ*E* ^total^ value of −11.15 kcal mol^−1^. It is stabilized by weak C–H···Cl interactions and further reinforced by π-stacking interactions between phenyl rings, with a separation of 3.681 (3) Å (Figure 6d). For this dimer, Δ*E* ^corr/disp^ contributes 75% to the stabilization. It is important to note that both chlorine atoms in this structure are involved in weak C–H···Cl hydrogen bonds. The analysis indicates that the combination of weak hydrogen bonds, chalcogen bonds, and π-stacking interactions collectively contribute to the stability and packing arrangement of compound **D** in the solid state.

### 3.4 Molecular dimers of compound E and their energetics

**FIGURE 7:**
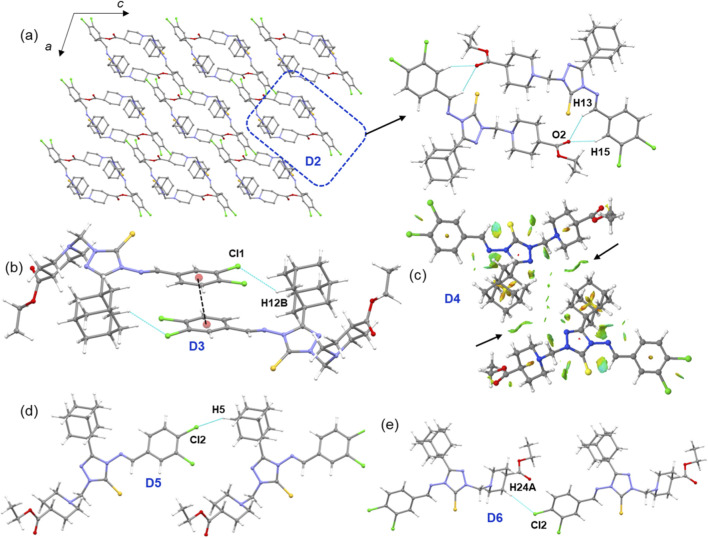
**(a)** Crystal packing of compound **E** projected onto the crystallographic *ac* plane, with the basic structural motif highlighted by a dashed box, and **(b–e)** molecular dimers observed in the crystal structure.

As shown in Figure 7a, molecules of compound **E** arrange in a columnar fashion, their specific arrangement and core structural motifs differ from those of compounds **B** and **D**. The crystal structure reveals six distinct molecular dimers, stabilized by various types of intermolecular interactions, with dimerization energies ranging from −20.97 to −1.69 kcal mol^−1^ (Table 4; Figures 7b–e). In contrast to compounds **B** and **D**, where the core structural motif is stabilized by short Csp^3^–H···H–Csp^3^ contacts, the basic structural motif in compound **E**, dimer D2, is stabilized by three-centered C–H···O interactions. This motif is the second-strongest dimer with a Δ*E* ^total^ value of −20.13 kcal mol^−1^. These C–H···O interactions are notably absent in compounds **B** and **D**. Energy decomposition analysis of D2 suggests that the electrostatic and dispersion energies contribute 56% and 44%, respectively, to its stabilization. This structure also lacks chalcogen bonds.

**TABLE 4:** Intermolecular interactions observed in various dimers of compound **E**, with decomposed energy components expressed in kcal mol^−1^.

Motif	Symmetry	Interactions	Geometry^a^ H···A (Å) ∠D–H···A ^a^°)	Δ*E* ^ele^	Δ*E* ^exrep^	Δ*E* ^pol^	Δ*E* ^corr/disp^	Δ*E* ^total^
D1	−*x*+1, −*y*+1, −*z*+2	C10–H10B···*Cg*1	2.69, 146	−6.06	12.34	−1.84	−25.40	−20.97
D2	−*x*+1, −*y*, −*z*+1	C13–H13···O2	2.56, 140	−17.03	22.11	−6.55	−18.66	−20.13
		C15–H15···O2	2.18, 155					
D3	−*x*+2, −*y*+1, −*z*+2	C12–H12B···Cl1	2.94, 136	−4.96	11.96	−1.61	−18.08	−12.68
		*Cg*1···*Cg*1	3.731 (2)					
D4	−*x*, −*y*+1, −*z*+1	Csp^3^–H···H–Csp^3^	<2.40	−2.08	6.39	−0.17	−11.35	−7.20
D5	*x*−1, *y*, *z*−1	C5–H5···Cl2	3.00, 127	−0.86	2.48	−0.13	−4.47	−2.98
D6	*x*−1, *y*−1, *z*−1	C24–H24A···Cl2	3.00, 156	−0.38	1.61	−0.35	−2.57	−1.69

^a^ Neutron diffraction values are given for all D–H⋅⋅⋅A interactions. *Cg*1 represents the centroid of the phenyl ring.

The strongest dimer, D1 (Δ*E* ^total^ = −20.97 kcal mol^−1^), is stabilized by intermolecular C–H···π interactions. In this interaction, the adamantane moiety acts as a donor, and the π-center of the dichloro phenyl ring serves as an acceptor. The NCI plot for D1 reveals large green patches between the interacting regions, confirming the presence of these weak C–H···π interactions (Supplementary Figure S3). Notably, dispersion energy contributes nearly 76% to the stabilization of this dimer. The D1 and D2 dimeric motifs are alternately interconnected to form a supramolecular chain that runs parallel to the crystallographic *c* axis.

The next most stable dimer, D3 (Δ*E* ^total^ = −12.68 kcal mol^−1^), is supported by a combination of a C–H···Cl interaction and π-stacking between adjacent dichloro phenyl rings, with a separation of 3.731 (2) Å. As expected, dispersion energy contributes 73% to its stabilization. A similar dimer is found in compound **D**, but the participating chlorine atom differs: in compound **D**, the ortho chlorine atom is involved, while in compound **E**, it is the meta chlorine atom. The D3 dimer in compound **E** is slightly stronger than its counterpart in compound **D**. Another recuring dimer, D4 (Δ*E* ^total^ = −7.20 kcal mol^−1^), is formed by short Csp^3^–H···H–Csp^3^ contacts between neighbouring adamantane groups. The NCI plot confirms the weak nature of these interactions, and energy decomposition analysis shows that dispersion energy contributes 83% to its stabilization. While the interaction energy of dimer D4 is nearly equal to its counterpart in compound **D**, the corresponding dimer in compound **B**, which is formed by similar Csp^3^–H···H–Csp^3^ contacts, exhibits even greater stability. The remaining two molecular dimers, D5 (Δ*E* ^total^ = −2.98 kcal mol^−1^) and D6 (Δ*E* ^total^ = −1.69 kcal mol^−1^), are stabilized by intermolecular C–H···Cl interactions. These interactions link the molecules into a chain in the solid state. Dispersion energy contributes 82% and 78%, to the stabilization of dimers D5 and D6, respectively.

### 3.5 Topological analysis of weak noncovalent interactions

The nature and strength of the intermolecular interactions governing the crystal packing of compounds B, D, and E were investigated using the QTAIM framework (Bader, 1991). The topological properties of the electron density, ρ(**r**), were analyzed at the bond critical points (BCPs) located between interacting atoms. Key topological parameters for all identified interactions in selected molecular dimers are summarized in Table 5, and the corresponding molecular graphs are illustrated in the Supplementary Material (Supplementary Figures S4-S6).

**TABLE 5:** Topological parameters for selected intermolecular interactions in different dimers of compounds **B**, **D** and **E**. *R*_ij_, Bond path (Å); *ρ*(**r**), Electron density (e Å^-3^); ∇^2^ *ρ*(**r**), Laplacian of electron density (e Å^-5^); *V*(**r**), Potential electron density (kJ mol^-1^ br^−3^); *G*(**r**), Kinetic electron density (kJ mol^-1^ br^−3^); *H*(**r**), Total electronic energy density (kJ mol^-1^ br^−3^); *D*_e_, Dissociation energy (kcal mol^-1^).

Interaction	*R*_ij_	*ρ*(r)	∇^2^ *ρ*(r)	*V*(r)	*G*(r)	*H*(r)	| $\frac{-V\left(r\right)}{G\left(r\right)}$ |	*D*_*e*_
Compound B								
D2								
H9···C13	2.815	0.042	0.429	−8.4	10.0	1.7	0.83	1.0
D3								
S1···N1	3.478	0.049	0.560	−9.9	12.6	2.7	0.79	1.2
D4								
S1···S1	3.524	0.053	0.584	−9.9	12.9	3.0	0.77	1.2
D5								
H17···N5	2.752	0.049	0.559	−10.4	12.8	2.4	0.81	1.2
D6								
H16···Cl1	3.001	0.034	0.384	−6.2	8.3	2.1	0.74	0.7
Compound D								
D1								
H24A···N2	2.683	0.049	0.537	−9.3	12.0	2.6	0.78	1.1
H19···Cl1	2.963	0.039	0.510	−7.9	10.9	3.0	0.73	1.0
D2								
H25B···S1	2.963	0.051	0.484	−8.9	11.1	2.1	0.81	1.1
S1···N1	3.210	0.068	0.901	−16.8	20.7	3.9	0.81	2.0
D3								
H12B···Cl2	2.982	0.039	0.446	−7.3	9.7	2.4	0.75	0.9
Compound E								
D2								
C15–H15···O2	2.204	0.100	1.489	−25.5	33.1	7.5	0.77	3.1
C13–H13···O2	2.583	0.047	0.641	−10.3	13.9	3.6	0.74	1.2
D3								
C12–H12B···Cl1	2.965	0.041	0.462	−7.7	10.1	2.5	0.76	0.9
D5								
H5···Cl2	3.036	0.036	0.431	−7.0	9.4	2.4	0.74	0.8
D6								
H24A···Cl2	3.023	0.033	0.371	−5.9	8.0	2.1	0.74	0.7

All interactions were characterized as closed-shell (non-covalent) in nature based on established criteria proposed by Gatti (Gatti, 2005) and Koch and Popelier (Koch and Popelier, 1995). This classification was confirmed by a positive value of the Laplacian of the electron density (∇^2^ρ(**r**) > 0), a positive total electronic energy density (H(**r**) > 0), and a ratio of the potential to kinetic energy density magnitudes of less than one (| $\frac{-V\left(r\right)}{G\left(r\right)}$ | < 1). The strength of these non-covalent contacts, particularly hydrogen bonds, referred to as the dissociation energy (*D*_e_), was estimated from the local potential energy density at the BCP using the relationship (*D*_e_ = ½ × *v*(**r**)), as formulated by Espinosa et al. (Espinosa et al., 1998). However, this relationship has also been extrapolated to provide reasonable estimates for other types of intermolecular interactions (Spackman, 2015; Tidey et al., 2018).

In compound **B**, the crystal packing is directed by a network of C–H···N, C–H···Cl, lp···π, and S···S chalcogen bonds. The estimated interaction energies are modest, ranging from 0.7 to 1.2 kcal mol-1. Within this network, the S···S chalcogen bond and the C–H···N interaction exhibits comparable strengths, while the C–H···Cl contact is significantly weaker. The packing in compound **D** is stabilized by a more diverse set of interactions. A relatively strong S···N chalcogen bond was identified, with a dissociation energy of 2.0 kcal mol^-1^. Its strength, which is nearly double that of the S···S bond in compound **B**, can be attributed to the greater directionality and electrostatic complementarity of the S···N contact. Additionally, a series of weaker C–H···N, C–H···S, and C–H···Cl hydrogen bonds contribute to the overall stability of compound **E**, with dissociation energy values ranging from 0.9 to 1.1 kcal mol^-1^. A C–H···O interaction was also present, exhibiting a strength comparable to the other hydrogen bonds in the structure.

### 3.6 Cytotoxicity assessment

The cytotoxicity compounds **A-E** was assessed against four human tumor cell lines including HepG-2 (hepatocellular carcinoma), MCF-7 (mammary gland breast cancer), HCT-116 (colorectal carcinoma), and PC-3 (human prostate cancer). The 3-[4,5-dimethylthiazoyl-2-yl]-2,5-diphenyltetrazolium bromide (MTT) colorimetric assay (Berridge and Tan, 1993; Mosmann, 1983) was used for the evaluation. Table 6 presents the results for compounds **A-E** and the reference anticancer drug doxorubicin (Tacar et al., 2013).

**TABLE 6:** Cytotoxicity of compounds A-E and the anticancer drug Doxorubicin against hepatocellular carcinoma (HepG-2), mammary gland breast cancer (MCF-7), colorectal carcinoma (HCT-116), and human prostate cancer (PC-3) cell lines.

Compound	X	In vitro cytotoxicity IC_50_ (µM)*			
		HepG-2	MCF-7	HCT-116	PC-3
A	2^a^F	22.20 ± 1.8	28.81 ± 2.0	52.48 ± 3.2	66.45 ± 3.2
B	2-Cl	12.64 ± 1.2	17.67 ± 1.0	67.42 ± 3.2	82.64 ± 4.1
C	2,6-F_2_	6.42 ± 1.1	8.60 ± 1.4	21.33 ± 1.4	26.04 ± 1.9
D	2,4-Cl_2_	38.40 ± 2.8	56.48 ± 3.0	>100	>100
E	3,4-Cl_2_	83.62 ± 5.1	>100	>100	>100
Doxorubicin		4.50 ± 0.2	4.17 ± 0.2	5.23 ± 0.3	8.87 ± 0.6

*IC_50_ < 25 (highly active), 26–5 (moderately active), 51–100 (weakly active), >100 (inactive).

The results indicate that the cytotoxicity of compounds **A-E** mainly depends on the nature and position of the halogen substituents (X) in the haloaryl moiety. The 2-fluorophenyl analogue **A** showed good activity against HePG-2, moderate activity against MCF-7, and weak activity against HCT-116 and PC-3 cell lines. Replacement of the fluorine atom in compound **A** with chlorine (compound **B**) enhanced the activity against HePG-2 and MCF-7 and altered the activity against HCT-116 and PC-3. The highest activity was observed for the 2,6-difluorophenyl analogue **C**, which exhibited potent activity against HePG-2, MCF-7, and HCT-116, and moderate activity against PC-3. In contrast, the 2,4-dichlorophenyl analogue **D** showed inferior activity compared to its 2-chlorophenyl analogue **B**, it retained only weak activity against HePG-2 and MCF-7 and lacked activity against HCT-116 and PC-3. Furthermore, replacing the 2,4-dichlorophenyl moiety in compound **D** with a 3,4-dichlorophenyl group (compound **E**) markedly reduced the activity; compound E retained only marginal activity against HePG-2 and showed no activity against MCF-7, HCT-116 and PC-3 cell lines.

### 3.7 Molecular docking analysis

Molecular docking was performed to explore the binding modes, interaction patterns, and affinities of compounds **A-E** with urokinase plasminogen activator (uPA), aiming to evaluate their potential as uPA inhibitors and to relate the findings to the *in vitro* data. The docking scores of compounds **A-C**, along with the control inhibitor, are listed in Table 7.

**TABLE 7:** Vina docking scores for the title compounds **A-C** and the co-crystallized inhibitor in kcal mol^-1^.

Compound	A	B	C	Co-crystallized inhibitor (AGB)
Vina docking score	−7.7	−8.2	−7.6	−6.8

**FIGURE 8:**
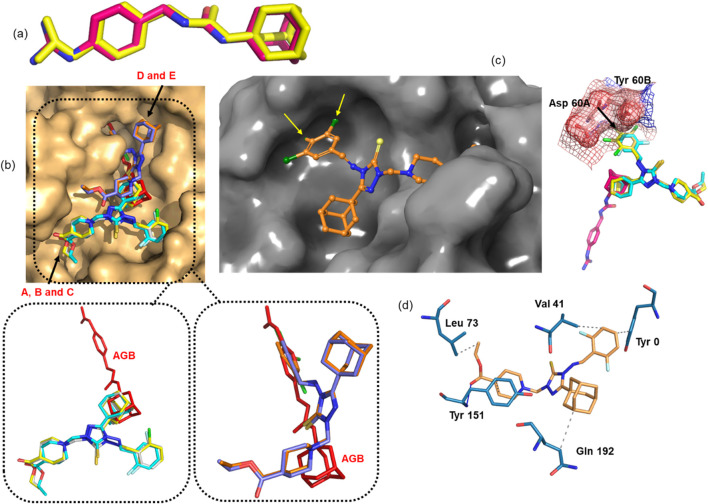
**(a)** Experimental (pink) and predicted (yellow) conformations of the control inhibitor (AGB), **(b)** surface representation of uPA showing the active-site cavity with the predicted conformations of compounds **A-E**; the dotted box highlights the similar orientations of compounds **A-C** and **D-E** along with the control inhibitor, **(c)** model of chlorine substitution at the 2,4,5 positions using the most potent compound **C**, showing steric hindrance with residues Asp 60A and Tyr 60B residues; surface and mesh representations further illustrate this steric hindrance, **(d)** uPA-compound C interactions.

The co-crystallized ligand *N*-(1-adamantyl)-*N*'-(4-guanidinobenzyl)urea (AGB), was first re-docked into uPA, showing excellent agreement between the predicted and crystallographic conformations, with a docking score of −6.8 kcal mol^-1^ (Figure 8a). Several docking poses of the title compounds (**A-E**) were carefully analyzed and selected based on the overlap of the adamantane ring between the control ligand (AGB) and the title compounds, as similar scaffolds are expected to occupy comparable positions within the active site. Interestingly, the most promising compounds **A-C** (against the HepG-2 cell line) obey this similarity principle, and their poses are nearly identical (Figure 8b). The docking scores of these three compounds range from −7.6 to −8.2 kcal mol^-1^, which are better than the control inhibitor, indicating comparable binding affinities that align well with the *in vitro* data.

In contrast, the docking poses of the lower-affinity dichloro compounds **D** and **E** differ markedly from those of compounds **A-C**, suggesting that their predicted poses may be unreliable. However, the predicted poses of **D** and **E** significantly overlap with that of the control inhibitor, without aligning their adamantane moieties, each showing a docking score of −7.1 kcal mol^-1^ (Figure 8b). According to the similarity principle, structurally similar compounds are expected to bind in similar orientations. To verify this, a simple modeling exercise was performed using the predicted pose of compound C (the most active compound), where a chlorine atom was substituted at the para position of the phenyl ring, and additionally at the 3,5-positions (considering the possible ring rotation) to mimic the 3,4-dichloro analog. As shown in Figure 8c, the 4-Cl substituent introduces steric hindrance with residues Asp 60A and Tyr 60B. Similarly, one orientation of the 3-Cl group is likely to clash sterically with Tyr 60B. This model clearly suggests that substitutions larger than a fluorine atom at the para position are prone to steric hindrance, which likely accounts for the reduced or complete loss of activity observed in such derivatives.

Since the predicted poses of the compounds **A-C** are very similar, the protein-ligand interaction analysis was carried out for the most active compound **C**. The analysis showed that the protein-ligand complex is primarily stabilized by hydrophobic interactions, in addition to hydrogen bonding (Figure 8d). The residues Val 41, Leu 73, Tyr 151, Gln 192 and the N-terminal Tyr 0 form hydrophobic interactions with compound C, while the backbone nitrogen of Tyr 151 also engages in a hydrogen bond with the carbonyl oxygen of the ligand.

### 3.8 Correlation between chemical descriptors, crystal packing features and *in vitro* cytotoxicity

The structures of compounds **A-E** were fully optimized according to the methods described in the experimental section. Vibrational frequency calculations confirmed that the optimized structures represent true energy minima on their respective potential energy surfaces, as indicated by the absence of imaginary frequencies. Furthermore, a structural superimposition of the optimized and X-ray conformations revealed a close resemblance, with root-mean-square deviation (RMSD) values ranging from 0.21 to 0.73 Å (Supplementary Figure S7). Minor structural distortions observed for compounds **D** and **E** are likely attributable to crystal packing effects.

**FIGURE 9:**
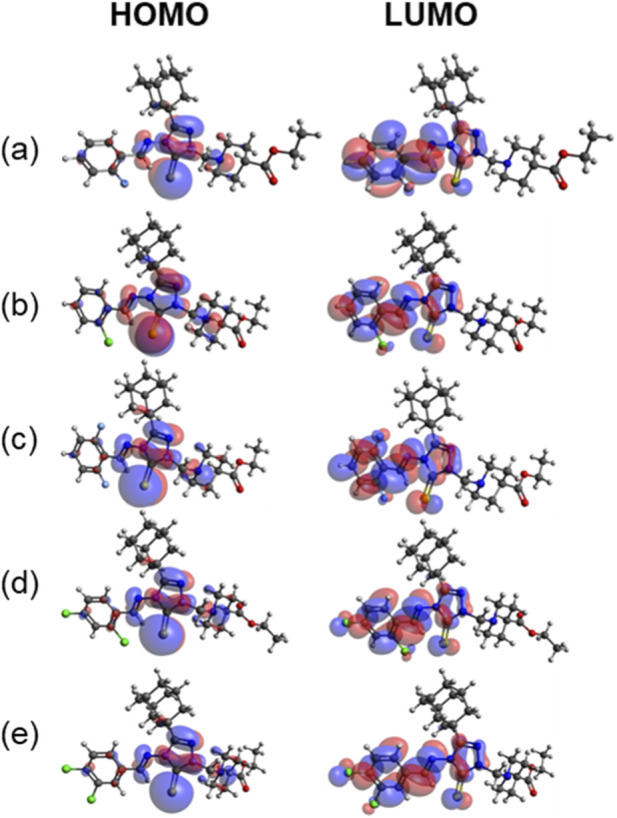
Highest occupied molecular orbitals (left panel) and lowest unoccupied molecular orbitals (right panel) for compounds **(a–e)**.

As shown in Figures 9a–e, the pattern of electron localization in the HOMO and LUMO orbitals is very similar irrespective of the substituents in the phenyl ring. In the HOMO orbitals, the electron densities are localized primarily over central triazole-3-thione moiety and over the nitrogen atoms. In contrast, the electron densities are localized primarily over the phenyl and triazole rings. The biological activity of a compound is intrinsically linked to its electronic properties, which dictate its reactivity and ability to interact with biological targets.

Recent review articles have shown that different studies use various molecular descriptors to predict the biological activity of small molecules (Huang et al., 2021; Vasilev and Atanasova, 2025). In this work, the relationship between the observed antiproliferative activity and key chemical properties of compounds **A-E** was examined by correlating their IC_50_ values (against the HepG-2 cell line) with selected physicochemical and electronic descriptors (Table 8). The descriptors included ADME (absorption, distribution, metabolism and excretion) parameters obtained from the SwissADME server (Daina et al., 2017) and quantum chemical indices such as HOMO and LUMO energies. Compounds showing IC_50_ values greater than 100 μM against other cell lines were considered weakly active and were excluded from the correlation analysis.

**TABLE 8:** Calculated molecular descriptors and biological activity (pIC50 = 6−log_10_(IC_50_) of Compounds **A-E**. Descriptors include quantum chemical indices (HOMO and LUMO energies in eV), lipophilicity (LogP), aqueous solubility (LogS), and Molar Refractivity.

Compound	pIC_50_ (HepG-2)	HOMO	LUMO	LogP	Log S	Molar refractivity
A	4.65	−6.873	−1.225	5.18	−6.14	147.91
B	4.90	−6.938	−1.266	5.34	−6.58	152.97
C	5.19	−6.937	−1.272	5.43	−6.31	147.87
D	4.42	−6.990	−1.460	5.88	−7.18	157.98
E	4.08	−7.049	−1.408	5.89	−7.18	157.98

As summarized in Table 8, the most active compound **C** exhibits a moderately lowest HOMO energy and the highest LUMO energy among the active molecules, resulting in a relatively small HOMO-LUMO energy gap. In contrast, less active dichloro derivatives **D** and **E** possess more negative HOMO energies, indicating reduced electron-donating ability and weaker interactions with the protein target. A moderate positive correlation between HOMO energy and pIC50 suggests that enhanced electron-donating capability facilitates strong donor-acceptor interactions at the binding site. The lipophilicity (log P) increases from 5.18 to 5.89, while the biological activity (pIC50) decreases from 4.65 to 4.08, revealing an inverse relationship. This suggests that excessive hydrophobicity may impair solubility or lead to steric hindrance within the active site, thereby reducing binding efficiency. Consistently, compounds A-C, which show higher activity, possess relatively greater solubility (log S = −6.1 to −6.6), whereas D and E are less soluble (log S = −7.18). This positive correlation between solubility and biological activity supports the notion that better aqueous compatibility enhances effective ligand-protein interactions. Moreover, compounds with moderate molar refractivity (148–153 cm^3^/mol; A-C) exhibit higher activity, while more polarizable analogues (D and E; 157.98 cm^3^/mol) show diminished potency. These observations suggest excessive molecular size and polarizability may hinder optimal accommodation of the ligand within the enzyme active site, leading to reduced binding affinity and lower cytotoxic activity.

**FIGURE 10:**
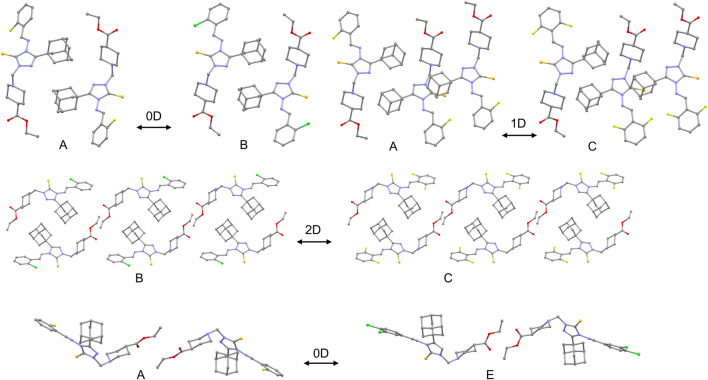
Structural similarity between different pairs of molecules.

To further explore the relationship between the observed activity and overall crystal packing features, an isostructurality analysis was carried out using the XPac 2.0 program (Gelbrich et al., 2012). This tool compares crystal structures based purely on the relative geometric conformations and spatial arrangements of molecules, without bias from perceived chemical effects such as specific intermolecular interactions. For this analysis, all available crystal structures, including the low-quality refined models of compounds A and C, were included. Although the crystal packing of compounds A-D is broadly similar, the degree of isostructurality varies among them (Figure 10). A 0D similarity (dimer match) is observed between A and B, while 1D (row of molecules match) and 2D (layer of molecule match) similarities are found between A-C and B-C pairs, respectively. The weakly or inactive compound E shares only 0D similarity with compound A, and no structural similarity is detected for other combinations. A notable packing feature common to the most active compounds (A-C) is the face-to-face stacking of adamantane moieties, stabilized by short H···H contacts, whose interaction energies are also significant. It is speculated that these short H···H contacts play an important role in the observed activity, whereas other intermolecular interactions in the crystal structures likely contribute marginally.

## 4 Conclusion

In this study, a series of five adamantyl-linked 1,2,4-triazole derivatives were synthesized, and their structures were characterized using single-crystal X-ray diffraction and computational methods. A detailed analysis of the crystal packing revealed that the supramolecular architecture of these compounds is governed by a diverse network of weak noncovalent interactions. Energy decomposition analysis confirmed that short Csp^3^–H···H–Csp^3^ contacts, chalcogen bonds (S···S and S···N), and various hydrogen bonds play a crucial role in the stabilization of molecular dimers, with dimerization energies ranging from −1.69 to −20.97 kcal mol^-1^. The relative contributions of these interactions were found to be dependent on the halogen substitution pattern on the phenyl ring. Molecular docking studies further rationalized the antiproliferative activity of these compounds by revealing favourable binding interactions with the active site of the urokinase plasminogen activator (uPA) enzyme. These findings emphasize the role of weak noncovalent interactions in guiding the rational design of bioactive molecules and identify this scaffold as a promising lead for uPA-targeted anticancer development. The observed variation in antiproliferative activity among the five compounds underscores the influence of the phenyl substituents (F or Cl at different positions), identifying this group as a key site for further optimization. This chemical scaffold thus represents a promising foundation for the rational design of new uPA-targeted anticancer agents.

## Data Availability

The supplementary crystallographic data could be obtained free of charge from Cambridge Crystallographic Data Centre (www.ccdc.cam.ac.uk/data_request/cif) using the accession numbers, CCDC-2447691 (A), CCDC-2447693 (B), CCDC-2447694 (C), CCDC-2447699 (D) and CCDC-2447700 (E).

## References

[B1] Adasme M. F. Linnemann K. L. Bolz S. N. Kaiser F. Salentin S. Haupt V. J. (2021). PLIP 2021: expanding the scope of the protein–ligand interaction profiler to DNA and RNA. Nucleic Acids Res. 49, W530–W534. 10.1093/nar/gkab294 33950214 PMC8262720

[B2] Aggarwal R. Sumran G. (2020). An insight on medicinal attributes of 1,2,4-triazoles. Eur. J. Med. Chem. 205, 112652. 10.1016/j.ejmech.2020.112652 32771798 PMC7384432

[B3] Ajmal M. Mahato A. K. Khan M. Rawat S. Husain A. Almalki E. B. (2024). Significance of triazole in medicinal chemistry: advancement in drug design, reward and biological activity. Chem. Biodivers. 21, e202400637. 10.1002/cbdv.202400637 38740555

[B4] Al-Omar M. A. Al-Abdullah E. S. Shehata I. A. Habib E. E. Ibrahim T. M. El-Emam A. A. (2010). Synthesis, antimicrobial, and anti-inflammatory activities of novel 5-(1-adamantyl)-4-arylideneamino-3-mercapto-1,2,4-triazoles and related derivatives. Molecules 15, 2526–2550. 10.3390/molecules15042526 20428062 PMC6257394

[B5] Al-Omary F. A. M. Chowdary Gude N. Al-Rasheed L. S. Alkahtani H. N. Hassan H. M. Al-Abdullah E. S. (2022). X-ray and theoretical investigation of (Z)-3-(adamantan-1-yl)-1-(phenyl or 3-chlorophenyl)-S-(4-bromobenzyl)isothioureas: an exploration involving weak non-covalent interactions, chemotherapeutic activities and QM/MM binding energy. J. Biomol. Struct. Dyn. 40, 2530–2545. 10.1080/07391102.2020.1840443 33150854

[B6] Al-Wahaibi L. H. Chakraborty K. Al-Shaalan N. H. Majeed M. Y. A. S. Blacque O. Al-Mutairi A. A. (2020). Quantitative analysis of hydrogen and chalcogen bonds in two pyrimidine-5-carbonitrile derivatives, potential DHFR inhibitors: an integrated crystallographic and theoretical study. RSC Adv. 10, 36806–36817. 10.1039/d0ra07215j 35517953 PMC9057057

[B7] Al-Wahaibi L. H. Grandhi D. S. Tawfik S. S. Al-Shaalan N. H. Elmorsy M. A. El-Emam A. A. (2021a). Probing the effect of halogen substituents (br, Cl, and F) on the non-covalent interactions in 1-(Adamantan-1-yl)-3-arylthiourea derivatives: a theoretical study. ACS Omega 6, 4816–4830. 10.1021/acsomega.0c05793 33644590 PMC7905817

[B8] Al-Wahaibi L. H. Rahul B. Mohamed A. A. B. Abdelbaky M. S. M. Garcia-Granda S. El-Emam A. A. (2021b). Supramolecular self-assembly built by weak hydrogen, chalcogen, and unorthodox nonbonded motifs in 4-(4-Chlorophenyl)-3-[(4-fluorobenzyl)sulfanyl]-5-(thiophen-2-yl)-4*H*-1,2,4-triazole, a selective COX-2 inhibitor: insights from X-ray and theoretical studies. ACS omega 6, 6996–7007. 10.1021/acsomega.0c06287 33748613 PMC7970574

[B9] Al-Wahaibi L. H. Alagappan K. Blacque O. Mohamed A. A. B. Hassan H. M. Percino M. J. (2022a). X-ray structures and computational studies of two bioactive 2-(Adamantane-1-carbonyl)-N-substituted Hydrazine-1-carbothioamides. Molecules 27, 8425. 10.3390/molecules27238425 36500517 PMC9741201

[B10] Al-Wahaibi L. H. Asokan K. V. Al-Shaalan N. H. Tawfik S. S. Hassan H. M. El-Emam A. A. (2022b). Supramolecular self-assembly mediated by multiple hydrogen bonds and the importance of C–S···N chalcogen bonds in N′-(Adamantan-2-ylidene)hydrazide derivatives. ACS Omega 7, 10608–10621. 10.1021/acsomega.2c00159 35382346 PMC8973099

[B11] Al-Wahaibi L. H. Macías M. A. Blacque O. Zondagh L. S. Joubert J. Thamotharan S. (2022c). Weak noncovalent interactions in three closely related adamantane-linked 1, 2, 4-Triazole N-Mannich bases: insights from energy frameworks, hirshfeld surface analysis, *in silico* 11β-HSD1 molecular docking and ADMET prediction. Molecules 27, 7403. 10.3390/molecules27217403 36364230 PMC9658560

[B12] Al-Wahaibi L. H. Mangaiyarkarasi S. Blacque O. M. Hassan H. El-Emam A. A. Percino M. J. (2023). Unusual short intramolecular N–H⋅⋅⋅H–C contact and weak intermolecular interactions in two N-(adamantan-1-yl)piperazine carbothioamides: crystallography, quantum chemical study and *in vitro* urease inhibitory activity. J. Mol. Struct. 1291, 136052. 10.1016/j.molstruc.2023.136052

[B13] Bader R. F. W. (1991). A quantum theory of molecular structure and its applications. Chem. Rev. 91, 893–928. 10.1021/cr00005a013

[B14] Bader R. F. W. (1994). Atoms in molecules: a quantum theory. USA: Oxford University Press.

[B15] Berridge M. V. Tan A. S. (1993). Characterization of the cellular reduction of 3-(4,5-dimethylthiazol-2-yl)-2,5-diphenyltetrazolium bromide (MTT): subcellular localization, substrate dependence, and involvement of mitochondrial electron transport in MTT reduction. Arch. Biochem. Biophys. 303, 474–482. 10.1006/abbi.1993.1311 8390225

[B16] Bicknell J. Agarwal S. A. Petersen K. J. Loya J. D. Lutz N. Sittinger P. M. (2024). Engineering Lipophilic Aggregation of Adapalene and Adamantane-Based Cocrystals *via* van der Waals Forces and Hydrogen Bonding. Cryst. Growth Des. 24, 5222–5230. 10.1021/acs.cgd.4c00457 38911135 PMC11191584

[B17] Bivacqua R. Barreca M. Spanò V. Raimondi M. V. Romeo I. Alcaro S. (2023). Insight into non-nucleoside triazole-based systems as viral polymerases inhibitors. Eur. J. Med. Chem. 249, 115136. 10.1016/j.ejmech.2023.115136 36708678

[B18] Britten C. D. Garrett-Mayer E. Chin S. H. Shirai K. Ogretmen B. Bentz T. A. (2017). A phase I study of ABC294640, a first-in-class sphingosine Kinase-2 inhibitor, in patients with advanced solid tumors. Clin. Cancer Res. 23, 4642–4650. 10.1158/1078-0432.CCR-16-2363 28420720 PMC5559328

[B19] Burla M. C. Caliandro R. Camalli M. Carrozzini B. Cascarano G. L. Giacovazzo C. (2012). SIR2011: a new package for crystal structure determination and refinement. J. Appl. Crystallogr. 45, 357–361. 10.1107/S0021889812001124

[B20] Chai J.-D. Head-Gordon M. (2008). Long-range corrected hybrid density functionals with damped atom–atom dispersion corrections. Phys. Chem. Chem. Phys. 10, 6615–6620. 10.1039/B810189B 18989472

[B21] Clark R. C. Reid J. S. (1995). The analytical calculation of absorption in multifaceted crystals. Acta Crystallogr. Sect. A 51, 887–897. 10.1107/S0108767395007367

[B22] Daina A. Michielin O. Zoete V. (2017). SwissADME: a free web tool to evaluate pharmacokinetics, drug-likeness and medicinal chemistry friendliness of small molecules. Sci. Rep. 7, 42717. 10.1038/srep42717 28256516 PMC5335600

[B23] Davies W. L. Grunert R. R. Haff R. F. Mcgahen J. W. Neumayer E. M. Paulshock M. (1964). Antiviral activity of 1-adamantanamine (amantadine). Sci. 144, 862–863. 10.1126/science.144.3620.862 14151624

[B24] Eberhardt J. Santos-Martins D. Tillack A. F. Forli S. (2021). AutoDock vina 1.2.0: new docking methods, expanded force field, and python bindings. J. Chem. Inf. Model. 61, 3891–3898. 10.1021/acs.jcim.1c00203 34278794 PMC10683950

[B25] El-Emam A. A. Saveeth Kumar E. Janani K. Al-Wahaibi L. H. Blacque O. El-Awady M. I. (2020). Quantitative assessment of the nature of noncovalent interactions in N-substituted-5-(adamantan-1-yl)-1,3,4-thiadiazole-2-amines: insights from crystallographic and QTAIM analysis. RSC Adv. 10, 9840–9853. 10.1039/D0RA00733A 35498588 PMC9050220

[B26] Espinosa E. Molins E. Lecomte C. (1998). Hydrogen bond strengths revealed by topological analyses of experimentally observed electron densities. Chem. Phys. Lett. 285, 170–173. 10.1016/S0009-2614(98)00036-0

[B27] Esterhuysen C. (2024). Hydrogen vs halogen-bonded R22(8) rings in organic crystal structures. Cryst. Growth Des. 24, 859–870. 10.1021/acs.cgd.3c01343

[B28] Frisch M. J. Trucks G. W. Schlegel H. B. Scuseria G. E. Robb M. A. Cheeseman J. R. (2013). Gaussian 09, revision D.01. Wallingford, CT, USA: Gaussian Inc.

[B29] Gao F. Wang T. Xiao J. Huang G. (2019). Antibacterial activity study of 1,2,4-triazole derivatives. Eur. J. Med. Chem. 173, 274–281. 10.1016/j.ejmech.2019.04.043 31009913

[B30] Gatti C. (2005). Chemical bonding in crystals: new directions. Z. für Krist. - Cryst. Mater. 220, 399–457. 10.1524/zkri.220.5.399.65073

[B31] Gelbrich T. Threlfall T. L. Hursthouse M. B. (2012). XPac dissimilarity parameters as quantitative descriptors of isostructurality: the case of fourteen 4,5′-substituted benzenesulfonamido-2-pyridines obtained by substituent interchange involving CF3/I/Br/Cl/F/Me/H. CrystEngComm 14, 5454–5464. 10.1039/C2CE25508A

[B32] Grimme S. Antony J. Ehrlich S. Krieg H. (2010). A consistent and accurate *ab initio* parametrization of density functional dispersion correction (DFT-D) for the 94 elements H-Pu. J. Chem. Phys. 132, 154104. 10.1063/1.3382344 20423165

[B33] Grimme S. Ehrlich S. Goerigk L. (2011). Effect of the damping function in dispersion corrected density functional theory. J. Comput. Chem. 32, 1456–1465. 10.1002/jcc.21759 21370243

[B34] Groom C. R. Bruno I. J. Lightfoot M. P. Ward S. C. (2016). The Cambridge structural database. Acta Crystallogr. Sect. B 72, 171–179. 10.1107/s2052520616003954 27048719 PMC4822653

[B35] Hehre W. J. Ditchfield R. Pople J. A. (1972). Self—consistent molecular orbital methods. XII. Further extensions of gaussian—type basis sets for use in molecular orbital studies of organic molecules. J. Chem. Phys. 56, 2257–2261. 10.1063/1.1677527

[B36] Huang T. Sun G. Zhao L. Zhang N. Zhong R. Peng Y. (2021). Quantitative structure-activity relationship (QSAR) studies on the toxic effects of nitroaromatic compounds (NACs): a systematic review. Int. J. Mol. Sci. 22, 8557. 10.3390/ijms22168557 34445263 PMC8395302

[B37] Keith T. A. (2019). AIMAll, ver. 19.02.13; TK gristmill software. Overland Park, KS.

[B38] Khan I. Panini P. Khan S. U.-D. Rana U. A. Andleeb H. Chopra D. (2016). Exploiting the role of molecular electrostatic potential, deformation density, topology, and energetics in the characterization of S···N and cl···N supramolecular motifs in crystalline triazolothiadiazoles. Cryst. Growth Des. 16, 1371–1386. 10.1021/acs.cgd.5b01499

[B39] Koch U. Popelier P. L. A. (1995). Characterization of C-H-O hydrogen bonds on the basis of the charge density. J. Phys. Chem. 99, 9747–9754. 10.1021/j100024a016

[B40] Liu J. Obando D. Liao V. Lifa T. Codd R. (2011). The many faces of the adamantyl group in drug design. Eur. J. Med. Chem. 46, 1949–1963. 10.1016/j.ejmech.2011.01.047 21354674

[B41] Liu Y. Yang X. Gan J. Chen S. Xiao Z.-X. Cao Y. (2022). CB-Dock2: improved protein–ligand blind docking by integrating cavity detection, docking and homologous template fitting. Nucleic Acids Res. 50, W159–W164. 10.1093/nar/gkac394 35609983 PMC9252749

[B42] Long J. Manchandia T. Ban K. Gao S. Miller C. Chandra J. (2007). Adaphostin cytoxicity in glioblastoma cells is ROS-Dependent and is accompanied by upregulation of heme oxygenase-1. Cancer Chemother. Pharmacol. 59, 527–535. 10.1007/s00280-006-0295-5 16924499

[B43] Milošev M. Z. Jakovljević K. Joksović M. D. Stanojković T. Matić I. Z. Perović M. (2017). Mannich bases of 1,2,4-triazole-3-thione containing adamantane moiety: synthesis, preliminary anticancer evaluation, and molecular modeling studies. Chem. Biol. Drug Des. 89, 943–952. 10.1111/cbdd.12920 27933733

[B44] Mosmann T. (1983). Rapid colorimetric assay for cellular growth and survival: application to proliferation and cytotoxicity assays. J. Immunol. Methods 65, 55–63. 10.1016/0022-1759(83)90303-4 6606682

[B45] Parthasarathi V. Kanagaraj H. (2024). A pharmacological update of triazole derivative: a review. Curr. Top. Med. Chem. 24, 2033–2049. 10.2174/0115680266308359240708094001 39069706

[B46] Protopopova M. Hanrahan C. Nikonenko B. Samala R. Chen P. Gearhart J. (2005). Identification of a new antitubercular drug candidate, SQ109, from a combinatorial library of 1,2-ethylenediamines. J. Antimicrob. Chemother. 56, 968–974. 10.1093/jac/dki319 16172107

[B47] Rathod B. Kumar K. (2022). Synthetic and medicinal perspective of 1,2,4-Triazole as anticancer agents. Chem. Biodivers. 19, e202200679. 10.1002/cbdv.202200679 36226542

[B48] Reuning U. Magdolen V. Wilhelm O. Fischer K. Lutz V. Graeff H. (1998). Multifunctional potential of the plasminogen activation system in tumor invasion and metastasis (review). Int. J. Oncol. 13, 893–906. 10.3892/ijo.13.5.893 9772277

[B49] Rosenthal K. S. Sokol M. S. Ingram R. L. Subramanian R. Fort R. C. (1982). Tromantadine: inhibitor of early and late events in Herpes simplex virus replication. Antimicrob. Agents Chemother. 22, 1031–1036. 10.1128/aac.22.6.1031 6297383 PMC185716

[B50] Sheldrick G. (2015). Crystal structure refinement with SHELXL. Acta Crystallogr. Sect. C 71, 3–8. 10.1107/s2053229614024218 25567568 PMC4294323

[B51] Spackman M. A. (2015). How reliable are intermolecular interaction energies estimated from topological analysis of experimental electron densities? Cryst. Growth Des. 15, 5624–5628. 10.1021/acs.cgd.5b01332

[B52] Spackman P. R. Turner M. J. McKinnon J. J. Wolff S. K. Grimwood D. J. Jayatilaka D. (2021). CrystalExplorer: a program for hirshfeld surface analysis, visualization and quantitative analysis of molecular crystals. J. Appl. Crystallogr. 54, 1006–1011. 10.1107/s1600576721002910 34188619 PMC8202033

[B53] Sperl S. Jacob U. Arroyo de Prada N. Stürzebecher J. Wilhelm O. G. Bode W. (2000). (4-Aminomethyl)phenylguanidine derivatives as nonpeptidic highly selective inhibitors of human urokinase. Proc. Natl. Acad. Sci. 97, 5113–5118. 10.1073/pnas.97.10.5113 10805774 PMC25790

[B54] Su P. Tang Z. Wu W. (2020). Generalized kohn-sham energy decomposition analysis and its applications. WIREs Comput. Mol. Sci. 10, e1460. 10.1002/wcms.1460

[B55] Tacar O. Sriamornsak P. Dass C. R. (2013). Doxorubicin: an update on anticancer molecular action, toxicity and novel drug delivery systems. J. Pharm. Pharmacol. 65, 157–170. 10.1111/j.2042-7158.2012.01567.x 23278683

[B56] Tang Z. Song Y. Zhang S. Wang W. Xu Y. Wu D. (2021). XEDA, a fast and multipurpose energy decomposition analysis program. J. Comput. Chem. 42, 2341–2351. 10.1002/jcc.26765 34626430

[B57] Thomas S. P. Dikundwar A. G. Sarkar S. Pavan M. S. Pal R. Hathwar V. R. (2022). The relevance of experimental charge density analysis in unraveling noncovalent interactions in molecular crystals. Molecules 27, 3690. 10.3390/molecules27123690 35744821 PMC9229234

[B58] Tidey J. P. Zhurov V. V. Gianopoulos C. G. Hermann T. S. Pinkerton A. A. (2018). QTAIM assessment of the Intra- and intermolecular bonding in a bis(nitramido–oxadiazolate) energetic ionic salt at 20 K. J. Phys. Chem. A 122, 9676–9687. 10.1021/acs.jpca.8b10065 30457862

[B59] Tratrat C. (2020). 1,2,4-Triazole: a privileged scaffold for the development of potent antifungal agents - a brief review. Curr. Top. Med. Chem. 20, 2235–2258. 10.2174/1568026620666200704140107 32621720

[B60] Vasilev B. Atanasova M. (2025). A (comprehensive) review of the application of quantitative structure–activity relationship (QSAR) in the prediction of new compounds with anti-breast cancer activity. Appl. Sci. 15, 1206. 10.3390/app15031206

[B61] Wanka L. Iqbal K. Schreiner P. R. (2013). The lipophilic bullet hits the targets: medicinal chemistry of adamantane derivatives. Chem. Rev. 113, 3516–3604. 10.1021/cr100264t 23432396 PMC3650105

[B62] Warda E. T. El-Ashmawy M. B. Habib E.-S. E. Abdelbaky M. S. M. Garcia-Granda S. Thamotharan S. (2022). Synthesis and *in vitro* antibacterial, antifungal, anti-proliferative activities of novel adamantane-containing thiazole compounds. Sci. Rep. 12, 21058. 10.1038/s41598-022-25390-0 36474013 PMC9726863

[B63] Weigend F. Ahlrichs R. (2005). Balanced basis sets of split valence, triple zeta valence and quadruple zeta valence quality for H to Rn: design and assessment of accuracy. Phys. Chem. Chem. Phys. 7, 3297–3305. 10.1039/B508541A 16240044

[B64] Xu Y. Zhang S. Wu W. Su P. (2023). Assessments of DFT-based energy decomposition analysis methods for intermolecular interactions. J. Chem. Phys. 158, 124116. 10.1063/5.0140912 37003781

[B65] Zhao Y. Truhlar D. G. (2008). The M06 suite of density functionals for main group thermochemistry, thermochemical kinetics, noncovalent interactions, excited states, and transition elements: two new functionals and systematic testing of four M06-class functionals and 12 other functionals. Theor. Chem. Acc. 120, 215–241. 10.1007/s00214-007-0310-x

