# Supplementary material for: Weak noncovalent interactions in 1,2,4-triazole-3-thione-linked adamantyl derivatives: experimental and computational insights into their potential as antiproliferative agents
Source: Front Chem. 2025 Nov 27;13:1691657. doi: 10.3389/fchem.2025.1691657 (PMC12703193; doi:10.3389/fchem.2025.1691657)
Supplement: Supplementary file 1 [file DataSheet1.docx]

**Supporting Information**

**Weak Noncovalent Interactions in 1,2,4-Triazole-3-Thione-Linked Adamantane Derivatives: Experimental and Computational Insights into Their Potential as Anti-Proliferative Agents**

Lamya H. Al-Wahaibi^1^, Annesha Chakraborty^2^, Hanan M. Hassan^3^, Mohammed S. M. Abdelbaky^4^, Santiago Garcia-Granda^5^, Ali A. El-Emam^6,^*, M. Judith Percino^7^ and Subbiah Thamotharan^2,^*

^1^ Department of Chemistry, College of Sciences, Princess Nourah bint Abdulrahman University, Riyadh 11671, Saudi Arabia

^2^ Biomolecular Crystallography Laboratory and DBT-Bioinformatics Center, School of Chemical and Biotechnology, SASTRA Deemed University, Thanjavur-613 401, India

^3^ Department of Pharmacology and Biochemistry, Faculty of Pharmacy, Delta University for Science and Technology, International Costal Road, Gamasa City, Mansoura 11152, Egypt

^4^ Department of Physical Chemistry, Faculty of Chemical Sciences, University of Salamanca, E-37008 Salamanca, Spain

^5^ Department of Physical and Analytical Chemistry, Faculty of Chemistry, Oviedo University-CINN, Oviedo 33006, Spain

^6^ Department of Medicinal Chemistry, Faculty of Pharmacy, Mansoura University, Mansoura 35516, Egypt

^7^ Unidad de Polímeros y Electrónica Orgánica, Instituto de Ciencias, Benemérita Universidad Autónoma de Puebla, Val3-Ecocampus Valsequillo, Independencia O2 Sur 50, San Pedro Zacachimalpa, Puebla 72960, CP, México

Table S1 Crystal data and refinement parameters for compounds **A** and **C**.

| Identification code | **A** | **C** |
| --- | --- | --- |
| Empirical formula | C_28_H_37_N_5_O_2_SF | C_28_H_34_N_5_O_2_SF_2_ |
| Formula weight | 525.68 | 542.66 |
| Temperature/K | 298(2) | 293(2) |
| Crystal system | triclinic | triclinic |
| Space group | *P*-1 | *P*-1 |
| a/Å | 7.2832(8) | 7.5346(13) |
| b/Å | 13.6617(13) | 13.728(3) |
| c/Å | 14.5348(16) | 14.257(3) |
| α/° | 104.011(9) | 101.665(15) |
| β/° | 95.894(9) | 97.373(14) |
| γ/° | 91.645(8) | 94.631(15) |
| Volume/Å^3^ | 1393.6(3) | 1423.6(5) |
| Z | 2 | 2 |
| ρ_calc_g/cm^3^ | 1.253 | 1.266 |
| μ/mm^‑1^ | 1.361 | 1.402 |
| F(000) | 562.0 | 574.0 |
| Crystal size/mm^3^ | 0.15 × 0.09 × 0.07 | 0.08 × 0.04 × 0.03 |
| Radiation | CuKα (λ = 1.54184) | CuKα (λ = 1.54184) |
| 2Θ range for data collection/° | 6.308 to 151.23 | 6.402 to 150.984 |
| Index ranges | -8 ≤ h ≤ 9, -17 ≤ k ≤ 17, -18 ≤ l ≤ 18 | -9 ≤ h ≤ 9, -17 ≤ k ≤ 17, -17 ≤ l ≤ 17 |
| Reflections collected | 27049 | 21370 |
| Independent reflections | 5791[R_int_ = 0.22, R_sigma_ = 0.1835] | 5825 [R_int_ = 0.3826, R_sigma_ = 0.4723] |
| Data/restraints/parameters | 5791/1/311 | 5825/0/343 |
| Goodness-of-fit on F^2^ | 1.023 | 0.849 |
| Final R indexes [I>=2σ (I)] | R_1_ = 0.0918, wR_2_ = 0.1402 | R_1_ = 0.0940, wR_2_ = 0.1253 |
| Final R indexes [all data] | R_1_ = 0.2418, wR_2_ = 0.2069 | R_1_ = 0.3805, wR_2_ = 0.2092 |
| Largest diff. peak/hole / e Å^-3^ | 0.84/-0.50 | 0.24/-0.20 |


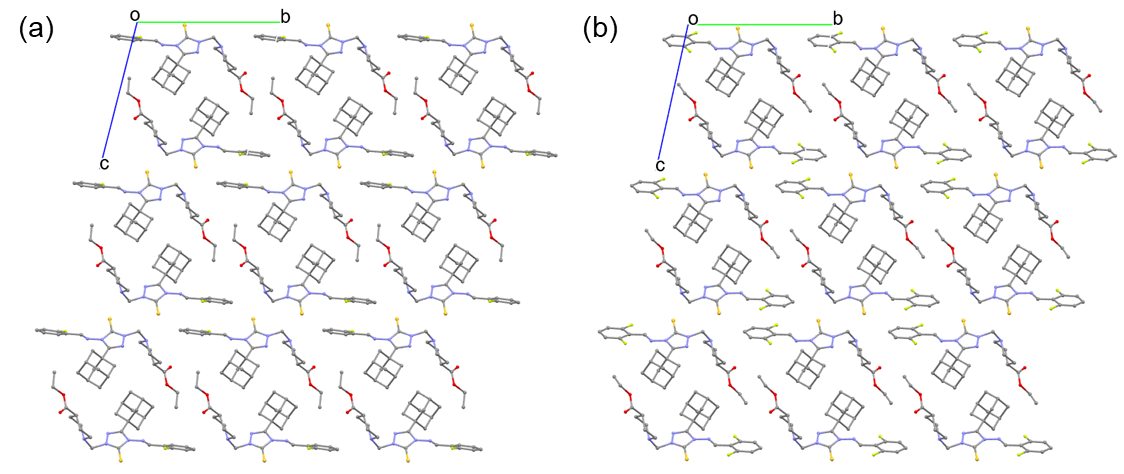


Figure S1 Crystal packing of compounds **A** (a) and **C** (b) projected onto the crystallographic *bc* plane.


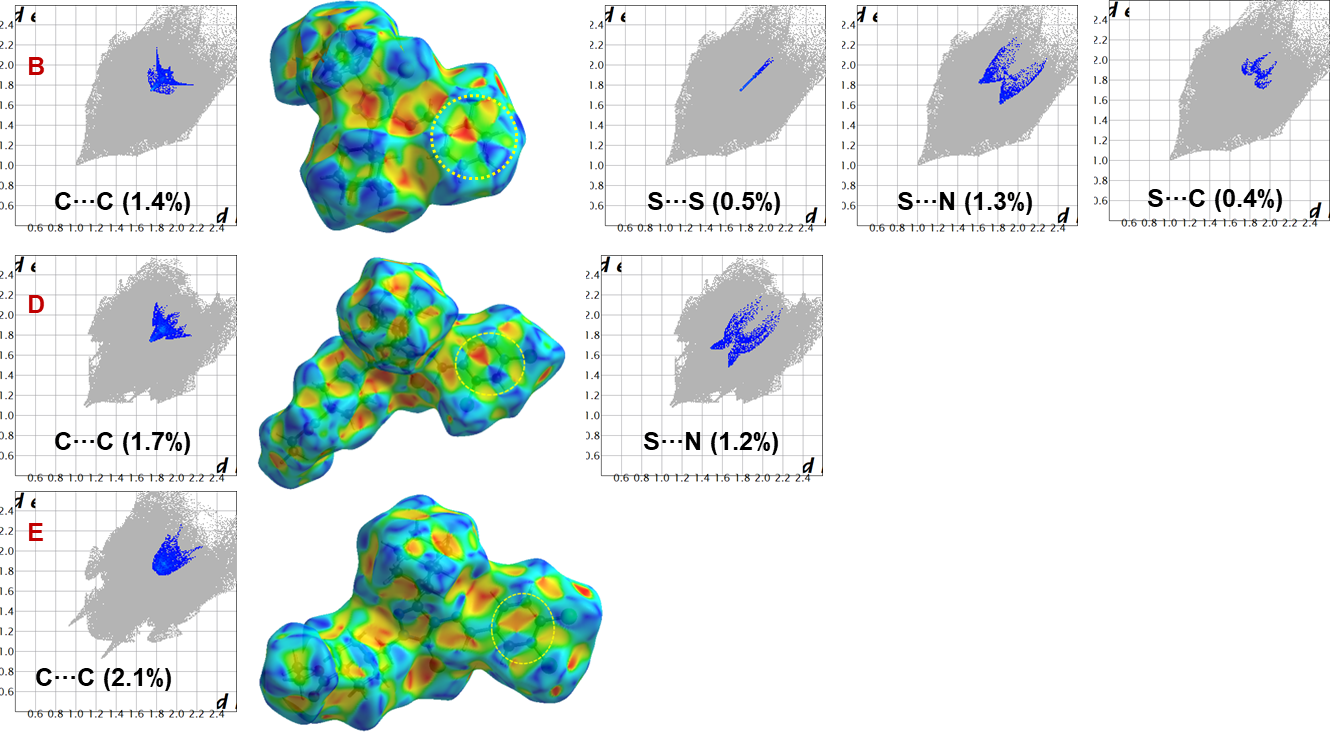


Figure S2 Selected decomposed fingerprint plots showing the percentage contributions for compounds **B**, **D** and **E**, along with the shape index diagram (red-blue triangles highlighted with dotted circles).


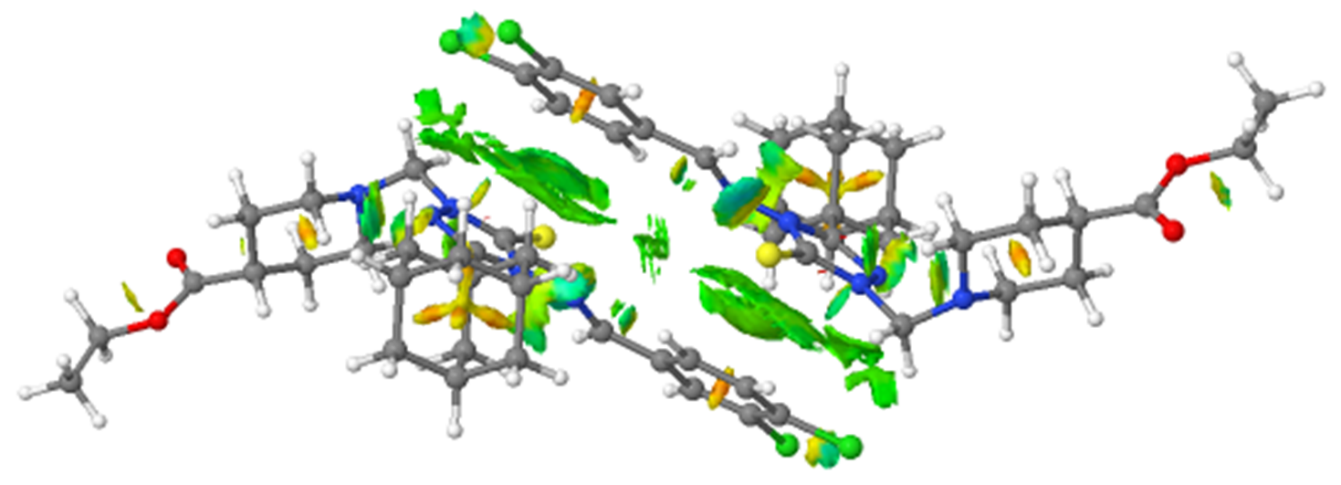


Figure S3 Molecular dimer D1 in compound E is stabilized by C–H···π interactions. The corresponding NCI plot confirm this weak attractive interaction, showing green patches in the interacting region.

Table S2 Topological parameters for selected intermolecular interactions in different dimers of compounds **B**, **D** and **E**. *R*_ij_, Bond path (Å); *ρ*(**r**), Electron density (e Å^-3^); ∇^2^*ρ*(**r**), Laplacian of electron density (e Å^-5^); *V*(**r**), Potential electron density (kJ mol^-1^ br^-3^); *G*(**r**), Kinetic electron density (kJ mol^-1^ br^-3^); *H*(**r**), Total electronic energy density (kJ mol^-1^ br^-3^); *D*_e_, Dissociation energy (kcal mol^-1^).

| Interaction | *R*_ij_ | *ρ*(**r**) | ∇^2^*ρ*(**r**) | *V*(**r**) | *G*(**r**) | *H*(**r**) | \|$\frac{-\boldsymbol{V}(\boldsymbol{r})}{\boldsymbol{G}(\boldsymbol{r})}$\| | *D_e_* |
| --- | --- | --- | --- | --- | --- | --- | --- | --- |
| **Compound B** | | | | | | | | |
| **D2** | | | | | | | | |
| H9···C13 | 2.815 | 0.042 | 0.429 | −8.4 | 10.0 | 1.7 | 0.83 | 1.0 |
| **D3** | | | | | | | | |
| S1···N1 | 3.478 | 0.049 | 0.560 | −9.9 | 12.6 | 2.7 | 0.79 | 1.2 |
| **D4** | | | | | | | | |
| S1···S1 | 3.524 | 0.053 | 0.584 | −9.9 | 12.9 | 3.0 | 0.77 | 1.2 |
| **D5** | | | | | | | | |
| H17···N5 | 2.752 | 0.049 | 0.559 | −10.4 | 12.8 | 2.4 | 0.81 | 1.2 |
| **D6** | | | | | | | | |
| H16···Cl1 | 3.001 | 0.034 | 0.384 | −6.2 | 8.3 | 2.1 | 0.74 | 0.7 |
| **Compound D** | | | | | | | | |
| **D1** | | | | | | | | |
| H24A···N2 | 2.683 | 0.049 | 0.537 | −9.3 | 12.0 | 2.6 | 0.78 | 1.1 |
| H19···Cl1 | 2.963 | 0.039 | 0.510 | −7.9 | 10.9 | 3.0 | 0.73 | 1.0 |
| **D2** | | | | | | | | |
| H25B···S1 | 2.963 | 0.051 | 0.484 | −8.9 | 11.1 | 2.1 | 0.81 | 1.1 |
| S1···N1 | 3.210 | 0.068 | 0.901 | −16.8 | 20.7 | 3.9 | 0.81 | 2.0 |
| **D3** | | | | | | | | |
| H12B···Cl2 | 2.982 | 0.039 | 0.446 | −7.3 | 9.7 | 2.4 | 0.75 | 0.9 |
| **Compound E** | | | | | | | | |
| **D2** | | | | | | | | |
| C15–H15···O2 | 2.204 | 0.100 | 1.489 | −25.5 | 33.1 | 7.5 | 0.77 | 3.1 |
| C13–H13···O2 | 2.583 | 0.047 | 0.641 | −10.3 | 13.9 | 3.6 | 0.74 | 1.2 |
| **D3** | | | | | | | | |
| C12–H12B···Cl1 | 2.965 | 0.041 | 0.462 | −7.7 | 10.1 | 2.5 | 0.76 | 0.9 |
| **D5** | | | | | | | | |
| H5···Cl2 | 3.036 | 0.036 | 0.431 | −7.0 | 9.4 | 2.4 | 0.74 | 0.8 |
| **D6** | | | | | | | | |
| H24A···Cl2 | 3.023 | 0.033 | 0.371 | −5.9 | 8.0 | 2.1 | 0.74 | 0.7 |


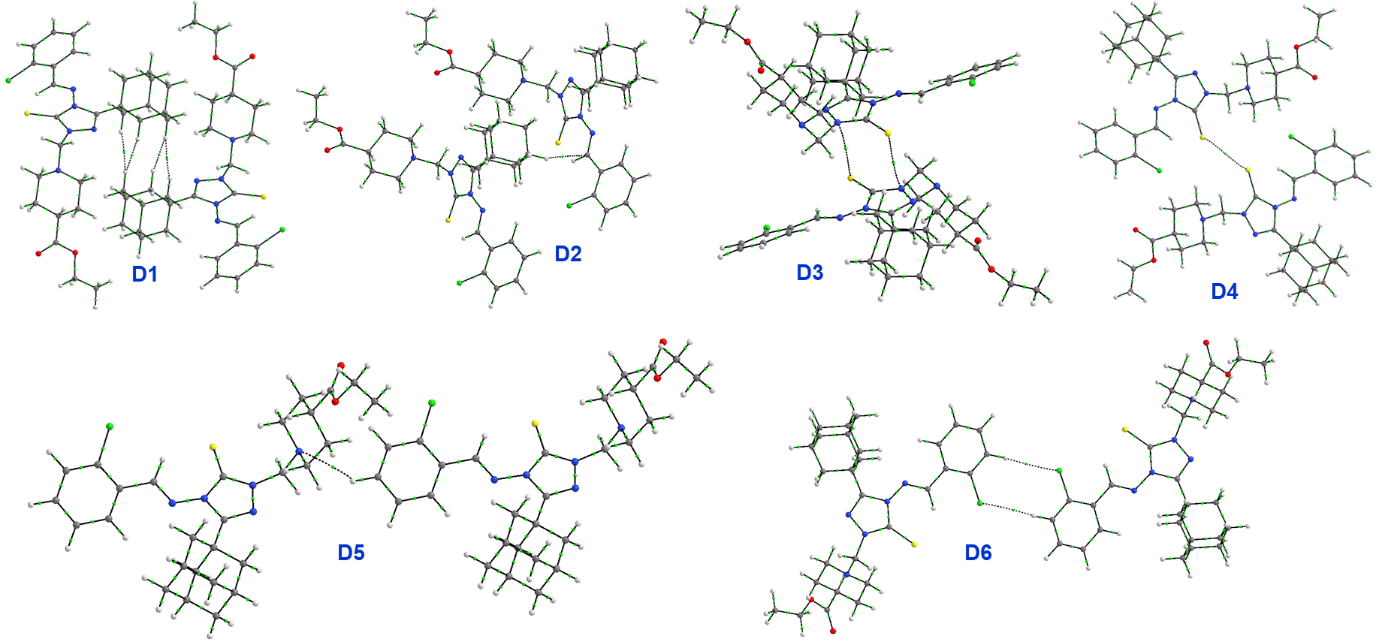


Figure S4 Molecular graphs showing the intermolecular contacts in various dimers of compound **B**.


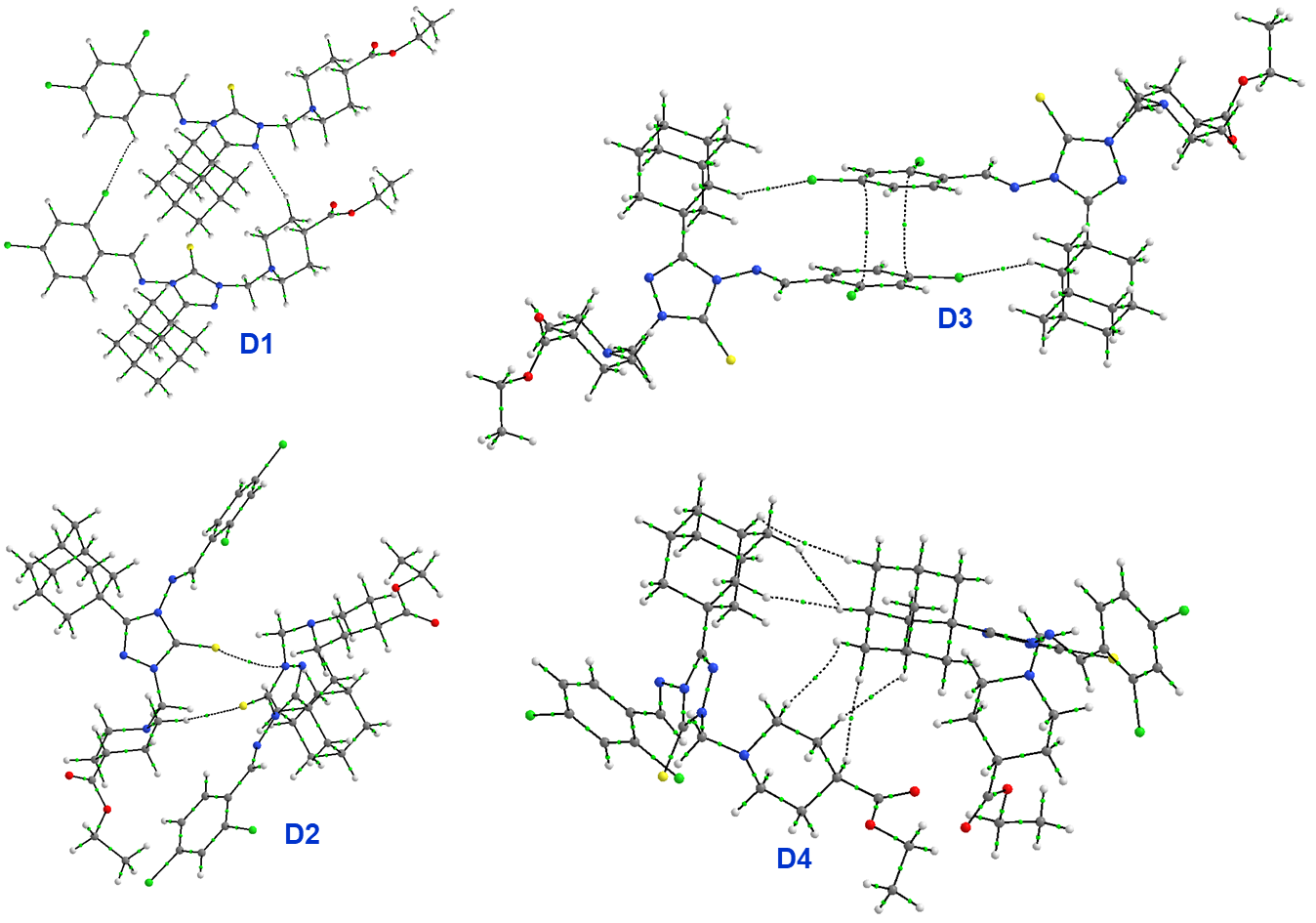


Figure S5 Molecular graphs showing the intermolecular contacts in various dimers of compound **D**.


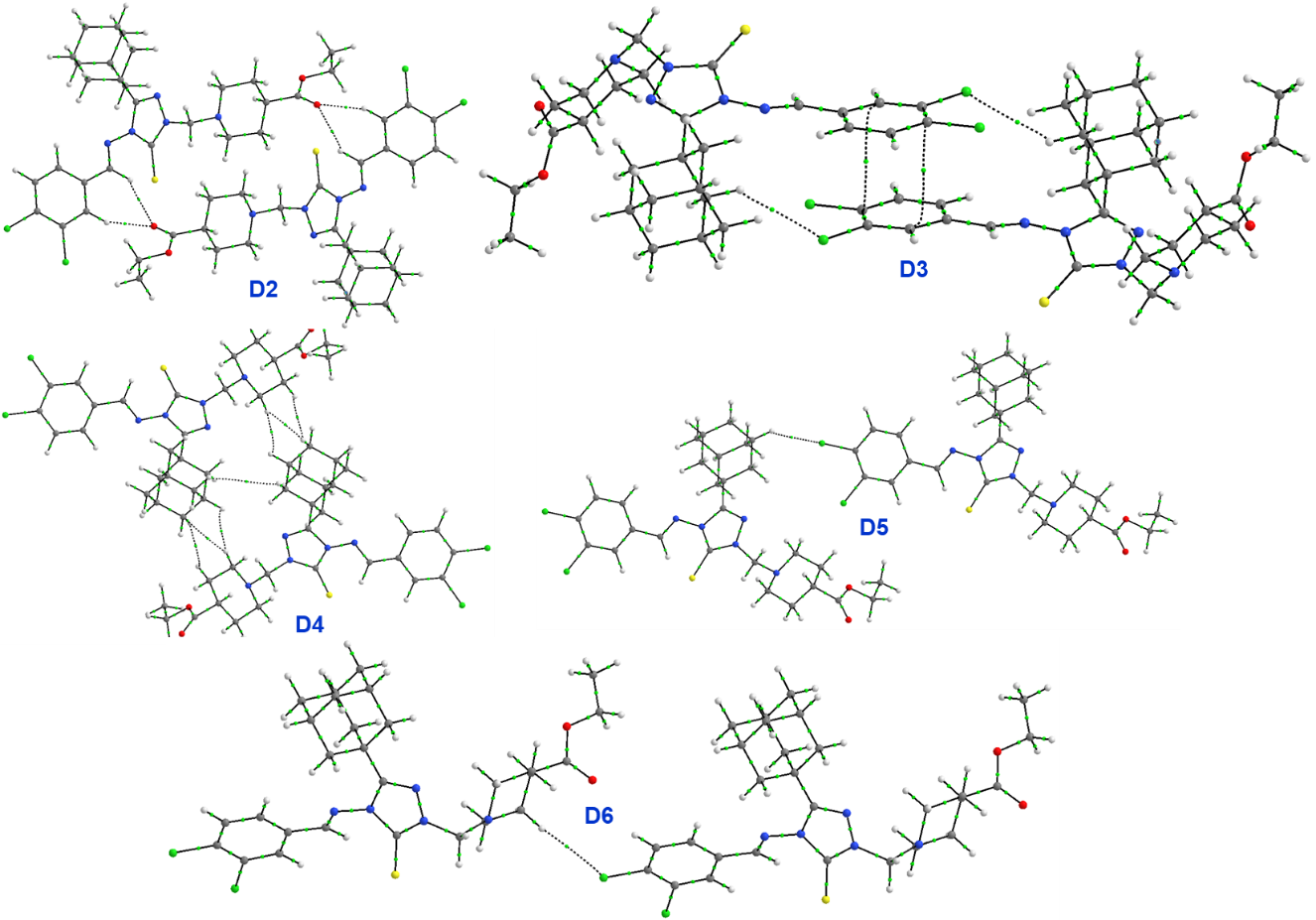


Figure S6 Molecular graphs showing the intermolecular contacts in various dimers of compound **E**.


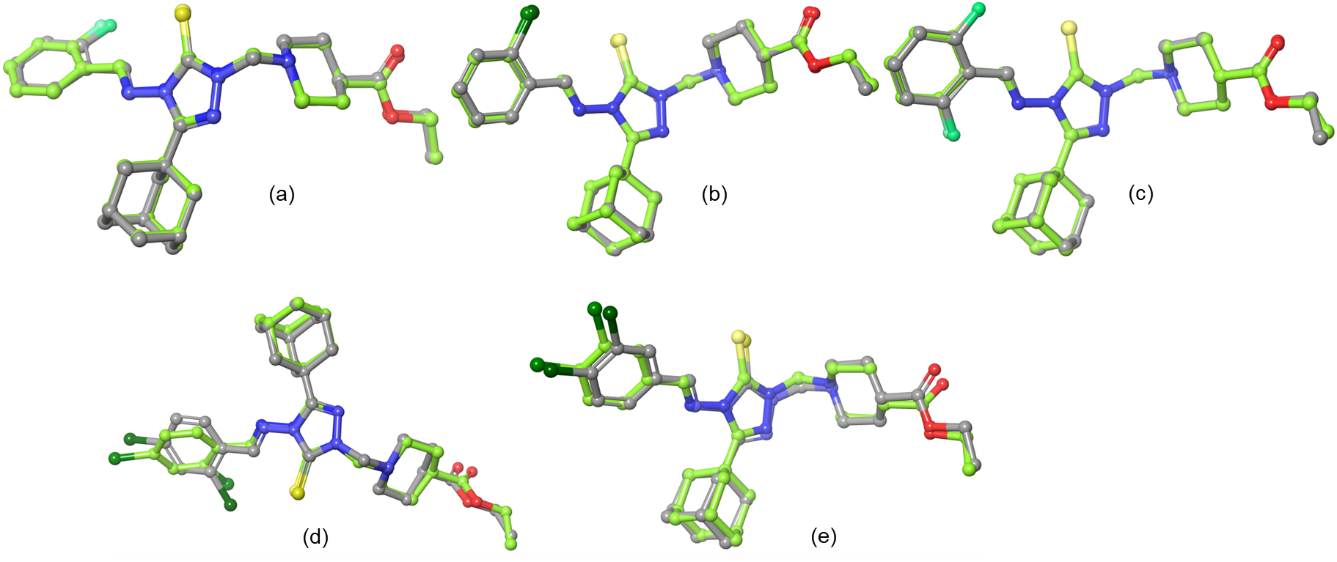


Figure S7 Structural superimposition of X-ray (green) and optimized (grey) structures of (a) compound **A**, (b) compound **B**, (c) compound **C**, (d) compound **D**, and (e) compound **E**.
